# Anaerobic Digestion of High-Solid Chicken Manure (CM) at Different Temperature: Intestinal Microbiome Efficiency, Inhibition, and Microbial Community Evolution

**DOI:** 10.3390/microorganisms13040724

**Published:** 2025-03-24

**Authors:** Xujing Chen, Qigui Niu, Jingyi Li, Zijing Zhou, Yue Wu, Guixue Song, Rutao Liu

**Affiliations:** 1 China-America CRC for Environment & Health, School of Environmental Science and Engineering, Shandong University, Qingdao 266237, China; 202222210066@mail.sdu.edu.cn (X.C.); 202012949@mail.sdu.edu.cn (J.L.); 202200210019@mail.sdu.edu.cn (Z.Z.); 202332960@mail.sdu.edu.cn (Y.W.); rutaoliu@sdu.edu.cn (R.L.); 2 Shandong Provincial Key Laboratory of Water Pollution Control and Resource Reuse, Shandong University, Qingdao 266237, China; 3 Shenzhen Research Institute, Shandong University, A301 Virtual University Park in South District, Shenzhen 518000, China; 4 Institute of Marine Science and Technology, Shandong University, Qingdao 266237, China; songgx@sdu.edu.cn

**Keywords:** high solid, Mono-CM digestion, inoculation, intestinal microbiome, microbial community succession

## Abstract

Anaerobic digestion (AD) of high-solid mono-chicken manure (CM) holds great promise for resource utilization. However, the effects of substrate overload (high-solid mixture inside the reactor) on AD performance at various temperatures are still unclear, moreover, the metabolic processes with and without inoculation are also seldom reported. In this study, three key impact factors of different temperatures (4 °C, 35 °C, 55 °C and 75 °C), total solids (TS) inside, and inoculation were conducted to comprehensively explore the process variation. EEM-FRI results revealed that high temps boost coenzyme F_420,_ while TS predominately driver the microbial production. High TS and temperature synthetically result in high free ammonia (FA) (>600 mg/L) associated with free volatile fatty acid (FVFA) (>450 mg/L), reducing CH_4_ production but increasing VFAs accumulation (12 g/L at 55 °C). Notably, intestinal microbiota alone without inoculation even achieved 11 g/L of VFA. The cross-feeding symbiosis between fermentative bacteria (*Caldicoprobacter*, *Bacteroidetes*, *Tepidimicrobium*) and hydrogenotrophic *Methanobacterium* enhanced CH_4_ production (68 mL/gVS at 35 °C). Moreover, high temperatures reduced microbial diversity but made heat-resistant hydrolytic bacteria dominant. This study precisely analyzes the effects of temperature and inoculation factors on the acidification efficiency of high-solid CM digestion, providing a crucial scientific basis for optimizing the resource utilization of CM waste.

## 1. Introduction

Globally, poultry production serves as a source of food for one billion people [1]. In China, around 146 million tons of chicken manure (CM) are produced per year [2,3]. Farming manure, being a globally abundant organic waste, can cause water eutrophication and contamination if not properly disposed of, raising serious environmental issues. Especially those extremely high-solid CM that have not been treated in a timely manner, or the large-scale accumulations caused by sudden situations [4].

Although numerous studies have shown that CM can be effectively treated through a series of pretreatment methods and anaerobic fermentation under optimized conditions [5,6,7,8,9]. In fact, the disposal of manure varies according to different scales of farming [10,11]. Particularly, the distinct temperatures in various seasons have a remarkable impact on the biotransformation that occurs during the storage and transportation of CM. Untreated high-solid CM, which is rich in ammonia, gives off a pungent odor. This not only attracts vermin and rodents but also spreads diseases, posing a serious threat to human health.

During the mono-anaerobic digestion (AD) of CM, high organic load rates trigger the hydrolysis of proteins, especially feeding with high total solids (TS), resulting in the overproduction of ammonia. This excess ammonia exerts both toxic and inhibitory influences on the activity of microorganisms involved in the digestion process [6]. Even with ammonia over 2000 mg/L, severe inhibition was observed in methanogenesis [8]. These challenges are exacerbated by seasonal temperature fluctuations, which significantly alter microbial consortia functionality and substrate bioavailability during storage and transportation. To counteract the detrimental effects of ammonia inhibition within anaerobic systems, a variety of strategies have been explored. These strategies mainly involve physical and chemical methods [3,4,8,12]. Nevertheless, these physical and chemical approaches are not only costly in terms of both financial resources and time investment but can also have a negative impact on AD, specifically, they may reduce microbial activity impeding the overall efficiency of AD. These combined effects ultimately lead to a decrease in digestion efficiency [13]. Although co-digestion and bioaugmentation strategies mitigate ammonia toxicity [4,6,7,14], their feasibility diminishes in large-scale, high-TS systems due to cost and operational complexity. Interestingly, the intrinsic metabolic potential of chicken intestinal microbiota-a self-contained hydrolytic community-remains unexplored as an alternative to engineered inocula [15]. The in vitro resource recovery driven by the intestinal microbiota of CM remains unclear. Moreover, the interplay between temperature, high TS (reaction in real), and microbial adaptation in CM lacks quantitative resolution, hindering the development of robust AD frameworks for poultry waste.

Thus, this study systematically investigates the impacts of temperature, TS, and inoculation on CM digestion, addressing three fundamental gaps. (1) How do temperature and inoculation extremes reshape microbial pathways (hydrolysis, acidogenesis, acetogenesis, and methanogenesis) under high-solid stress? (2) What are the quantitative boundaries for free ammonia (FA)/free VFA (FVFA) accumulation that trigger process failure? This work provides actionable insights for managing high-solid waste in variable temperatures.

## 2. Materials and Methods

### 2.1. Feedstock, Inoculum and Operating Procedure

The inoculum sludge was taken from a citric acid wastewater treatment facility in Shandong Province, China. The anaerobic batch tests were carried out under psychrophilic conditions (4 ± 0.5 °C), mesophilic conditions (35 ± 1 °C), thermophilic conditions (55 ± 1 °C), and super thermophilic conditions (75 ± 1 °C), respectively. The original CM was taken from a broiler-producing poultry farm in Qingdao City, Shandong Province, China. The TS and VS were 16% and 76%, respectively. The original CM was used directly without any pretreatment. All batch tests were standardized with a consistent F/M ratio to ensure a relatively stable basis for comparison (see Supplementary Materials). Furthermore, tests with different doses of reactional TS were conducted to evaluate the influence of TS on anaerobic digestion performance (Table S1).

### 2.2. Chemical and Physiological Analysis Procedures

The chemical indicators, including pH, TS, chemical oxygen demand (COD), NH_4_^+^, and volatile solid (VS), were analyzed in strict accordance with the Standard Methods detailed in our previous report [6]. The gas yields were measured using a vacuum glass syringe, and the biogas composition was analyzed by a GC136 gas chromatograph equipped with a TDX chromatographic column (packed column) and TCD detector (INESA, Shanghai, China). The H_2_, N_2_, CH_4_, and CO_2_ contents were analyzed based on the calibration of the standard curves. The VFAs were determined by High-Performance Liquid Chromatography (HPLC2030, Simadazi, Kyoto, Japan) [16].

### 2.3. Extracellular Polymeric Substances (EPS) Extraction and Analysis

EPS were extracted by the ‘modified heat method’ as described in our previous report [6]. For each sample, the three kinds of soluble microbial production EPS (SMP), loosely bound EPS (LB-EPS), and tightly bound EPS (TB-EPS) were obtained. The total protein (PN) and the polysaccharides (PS) of each EPS were detected at different temperatures (T) Moreover, the Excitation-Emission Matrix (EEM) for Fluorescence Regional Integration (EEM-FRI) was analyzed using a F-4600 Fluorescence Spectrophotometer (Hitachi, Kyoto, Japan) based on our previous description [5,17].

### 2.4. Kinetic Analysis

The following modified Gompertz model was used for biogas simulation [8].

(1) $$P_{CH4}(t)=P\times \exp[-\exp(\frac{K_{max}\times 2.73}{P(A-t)}+1)]$$

where, P is the maximum methane potential (suitable for biogas and bio CO_2_), in mL/gVS; K is the maximum methane production rate, in mL/(gVS/h); *A* is the lag time, in hours; t is the response time, in hours.

The Hydrolysis, Acidogenesis, Acetogenesis, and Methanogenesis were calculated based on the COD conversion as described in our previous research [8].

(2) $$\mathrm{Hydrolysis}=\frac{\left({TCOD}_{in}-{SCOD}_{in}\right)-({TCOD}_{out}-{SCOD}_{out})}{{TCOD}_{in}-{SCOD}_{in}}$$

(3) $$\mathrm{Acidogenesis}=\frac{\left({COD}_{{VFA}_{out}}-{COD}_{{VFA}_{in}}+{COD}_{{CH}_{4}}+{COD}_{H_{2}}\right)}{{TCOD}_{in}-{COD}_{{VFA}_{in}}}$$

(4) $$\mathrm{Acetogenesis}=\frac{\left({COD}_{{HAc}_{out}}-{COD}_{{HAc}_{in}}+{COD}_{{CH}_{4}}+{COD}_{H_{2}}\right)}{{TCOD}_{in}-{COD}_{{HAc}_{in}}}$$

(5) $$\mathrm{Methanogenesis}=\frac{\left({COD}_{{CH}_{4}}+{COD}_{H_{2}}\right)}{{TCOD}_{in}}$$

### 2.5. Free VFA (FVFA) and Free Ammonia (FA) Calculation

Free VFA was calculated as the following equilibrium expression [8]:

(6) $$\mathrm{Free}\;\mathrm{VFA}=\frac{VFA*10^{\left(pka-pH\right)}}{1+10^{\left(pka-pH\right)}}$$

where: VFA is the VFA concentration in digestion; pKa was the dissociation constants of the individual VFAs, with values of 4.757, 4.874, and 4.812 for acetic, propionic, and butyric acids at 25 °C, respectively [8].

(7) $$\frac{{NH}_{3}}{TAN}={\left(1+\frac{10^{-pH}}{10^{\left(0.09018+\frac{2729.92}{T(k)}\right)}}\right)}^{-1}$$

The free ammonia was calculated based on Equation (7), Where TAN is the total ammonia concentration.

### 2.6. DNA Extraction and Sequencing

The total genomic DNA of selected batch samples was extracted by using PowerSoil^®^ DNA Isolation Kit (QIAGEN, Hilden, Germany) according to the manufacturer’s protocols. Samples were washed triply with PBS buffer and centrifuged (12,000× *g*) for 3 min at 4 °C. DNA concentration was measured using nanodrop. The accurate DNA concentration of 20–30 ng DNA was used to generate amplicons. The V3 and V4 hypervariable regions of prokaryotic 16S rDNA were selected for generating amplicons by a pair of specifically designed primers (5′−3′: “CCTACGGRRBGCASCAGKVRVGAAT”; 3′−5′: GGACTACNVGGGTWTCTAATCC”). Thermocycling began at 95 °C for 3 min followed by 24 cycles of 5 s at 9 °C, 90 s at 57 °C and 10 s at 72. A final extension step of 72 °C for 5 min was also conducted. The DNA libraries were multiplexed and loaded on an Illumina MiSeq instrument according to the manufacturer’s instructions (Illumina, San Diego, CA, USA) by GENEWIZ^®^. All of the sequencing reads were filtered based on the sequence length being greater than 200 bp, and all of the reads were processed using Qiime 2 (v. 2024.10) to obtain the taxonomy by referencing NCBI. Moreover, the package of “Tax4Fun2” was applied to the community functional prediction with the support of Kyoto Encyclopedia of Gene and Genome (KEGG) database (https://www.genome.jp/kegg/pathway.html, accessed on 1 January 2025). General information on functional pathways and prospected KO (KEGG Orthology) was performed to reveal the metabolic response at the level of the microbial community.

### 2.7. Statistical Analysis and Functional Prediction

The results are expressed as the mean ± standard deviation for each experiment with duplicated analysis. Data normality was assessed using the Shapiro–Wilk test. For parametric data, significant differences were determined by a parametric one-way ANOVA test followed by a Tukey post-hoc test (*p* < 0.05). Alpha diversity indicators such as the Shannon index, Simpson index, Chao, and ACE were calculated in R, and the PCA, redundancy analysis (RDA), and mental analysis were generated in R 4.4.2 (November 2024) with a significant level of Pearson’r > 0.8, *p* < 0.05 or Spearman’r > 0.8, *p* < 0.05 (package “psych”, “vegan” November 2024).

## 3. Results and Discussion

### 3.1. Biogas Performance Response to TS and Temperature Variation

**Figure 1:**
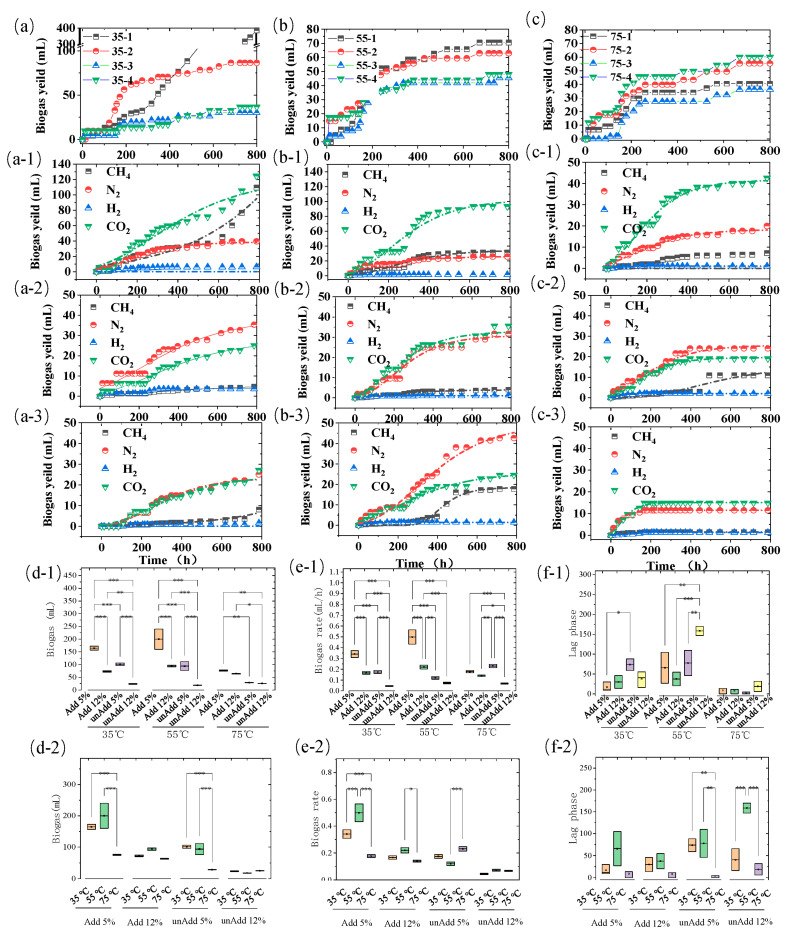
(**a**–**c**,**a-1**–**c-3**) Cumulative biogas yield with CH_4_, N_2_, H_2_, and CO_2_ components overtime at 35 °C, 55 °C, and 75 °C; biogas yield under different temperatures (**d-1**) and TS concentration conditions (**d-2**); biogas production rate under different temperatures (**e-1**) and TS concentration conditions (**e-2**); lag phase duration under different temperature (**f-1**) and TS concentration conditions (**f-2**). * represents *p* < 0.05, ** represents *p* < 0.01, *** represents *p* < 0.001.

The physicochemical characteristics of organic substrates, particularly total solids content and temperature sensitivity, critically influence the metabolic pathways and process stability in anaerobic digestion systems. As demonstrated in Figure 1a–c, our comparative analysis of biogas production under psychrophilic (4 ± 0.5 °C), mesophilic (35 ± 1 °C), thermophilic (55 °C), and super-thermophilic (75 °C) conditions revealed temperature-dependent transitions in microbial consortia functionality.

At psychrophilic conditions (4 °C), methanogenic archaea entered a metabolically dormant state, completely arresting substrate conversion to methane (CH_4_) or carbon dioxide (CO_2_). This metabolic shutdown aligns with previous reports of thermal deactivation thresholds for aceticlastic methanogens below 10 °C [18]. In contrast, mesophilic systems exhibited optimal biomethane yields, with inoculated reactors achieving 160 mL cumulative CH_4_ (68 mL/gVS) at 800 h—threefold higher than non-inoculated controls (Figure 1a). The critical TS threshold emerged at 5%, beyond which gas production exhibited inverse proportionality to solids loading. Specifically, 12% TS reactors showed a 98.6% reduction in CH_4_ yield (0.92 vs. 68 mL/gVS) compared to 5% TS systems, likely due to mass transfer limitations and volatile fatty acid accumulation.

Notably, thermophilic regimes displayed complete methanogenesis inhibition in non-inoculated systems, while CO_2_ production persisted at 4.03 mL/gVS (5% TS) and 1.29 mL/gVS (10% TS) at 55 °C. This suggests partial retention of hydrolytic/acidogenic activity despite methanogen inactivation. The observed biohydrogen production (2.84 mL/gVS under 55 °C/10% TS) implies redirected electron flow towards hydrogenotrophic pathways under thermal stress, consistent with enhanced hydrogenase expression patterns reported in extreme environments [18].

**Figure 2:**
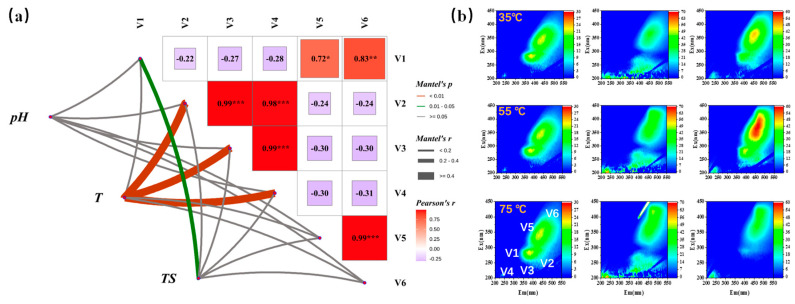
Mental analysis for the key operational factors (**a**) and the EEM-FRI of DOM associated with different temperatures (**b**). * represents *p* < 0.05, ** represents *p* < 0.01, *** represents *p* < 0.001.

The interplay between TS concentration and operational temperature exhibited profound impacts on biogas generation dynamics, as systematically quantified in Figure 1(d-1–f-2). A similar trend was also noted in the comparison of the biogas production rate. Under mesophilic conditions, when combined with a low level of reaction TS, the most favorable outcome can be achieved when methanogens are inoculated. Nevertheless, even in the absence of methanogens and with only the intestinal microbiota present, CM can still undergo efficient biochemical treatment and generate an equivalent quantity of carbon dioxide (Figure 1a–c). This further substantiates the activity of the microbiota and the feasibility of degradation.

Our results are contrary to those of previous reports [19], which reported a high biomethane production of 240 mL/gVS under an organic loading rate (OLR) of 2.5 kg VS/m^3^/d. These values are significantly lower than the results obtained of 540 mL CH_4_/gVS [14]. However, the organic loading rate is 3 gVS/L/d with very low reactional TS and employed in the form of co-digestion with low ammonia concentration [20]. High-solid CM digestion performance suggests that substrate composition TS inside reaction and operational temperature exert greater influence on methanogenesis than loading rate parameters alone.

### 3.2. The Influence of TS and Temperature on Dissolved Organic Matter (DOM)

The EEM-FRI of DOM associated with different temperatures are illustrated in Figure 2. The soluble microbial by-product-like substances (SMP) V1 were measured at Ex 250–340 nm and Em 280–380 nm, the fulvic acid-like substances V2 were measured at Ex 220–250 nm and Em 380–500 nm, the tryptophan-like substances V3 were measured at Ex 220–250 nm and Em 330–380 nm, the tyrosine-like substances V4 were measured at Ex 220–250 nm and Em 280–330 nm, the humic acid-like substances V5 were measured at Ex 250–400 nm and Em 380–500 nm, and the coenzyme 420 of V6 was measured at Ex/Em of 420/470 (Figure 2b) [21]. In this study, high temperatures led to a high concentration of the cofactor F420 (V6), which prompted cells to secrete more enzymes extracellularly. Additionally, there is a close correlation between the abundances of V1 and V6, which are attributed to microbial abundance. V1 represents the activity of microbial individuals, particularly hydrogenotrophic methanogens. Moreover, the cofactor F420 was wildly detected in many kinds of bacteria, and may confer several competitive advantages by mediating their central metabolic processes [22], indicating the more complex metabolic.

Temperature significantly influences the DOM variation of V2, V3, and V4 (*p* < 0.05), while TS significantly influences the V1 variation (Figure 2a). However, the pH maintains a stable level (Table S2) and has no significant effect on DOM. V3 tyrosine and V4 tryptophan are closely related, suggesting that V3 and V4 are strongly associated with microbial metabolic activity, which further confirms the significant impact of temperature on the bacterial protein secretion system. During the reaction, the contents of V5 and V6 increase significantly (Figure S1), indicating an improvement in microbial metabolic efficiency and a tendency for the system to stabilize.

### 3.3. The Influence of TS and Temperature on VFAs

**Figure 3:**
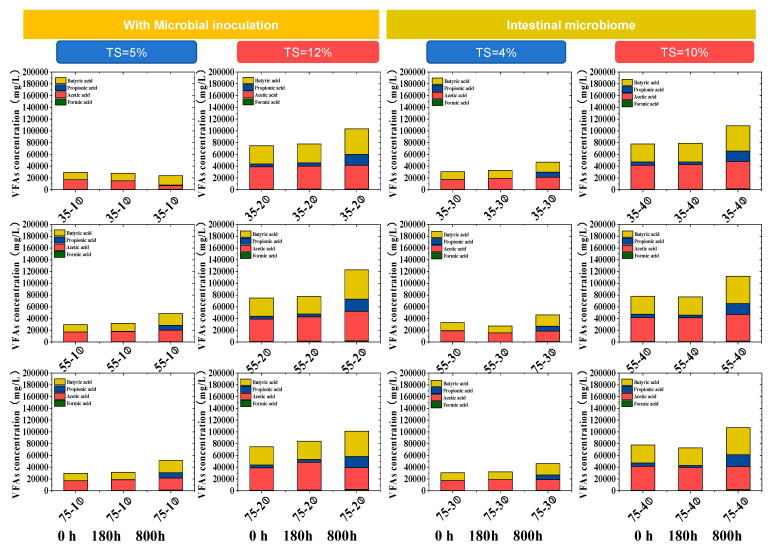
Influence of temperature on dominate VFAs variation of digestion process following opertation time. ① represents the sampling time of 0 h, ② represents the sampling time of 180 h, ③ represents the sampling time of 800 h.

Single-stage anaerobic digestion encounters the problem of poor system stability. This issue becomes even more prominent when dealing with substrates of high organic load or those that are easily biodegradable [23,24]. The individual VFAs of all tests following the time series (Figure 3) clearly demonstrated that the solid content has a significant impact on the production of volatile fatty acids (VFA). For the experimental groups inoculated with sludge, under the environmental condition of 35 °C, the content of volatile fatty acids (VFA) in the 35-1 test showed a significant decrease at 800 h. This change trend is highly consistent with the gas-production phenomenon of a large amount of methane and carbon dioxide during this period (Figure 1). However, under the conditions of 55 °C and 75 °C, the VFA concentration exhibited a distinct upward trend, reaching a maximum of 12 g/L. In the experimental group without sludge inoculation, which only contains chicken intestinal flora, the VFA concentration can also reach a maximum of 11 g/L. This fully indicates that the chicken intestinal microorganisms themselves possess strong acidification activity, while the contribution of inoculated sludge to acid production is not remarkable. However, under the 75 °C condition, it is hypothesized that thermophilic flora with strong adaptability might be present. This thermophilic flora can not only sustain the acid-production efficiency but also potentially enhance it further. This discovery provides a brand—new research direction for in-depth exploration of the field of high-temperature fermentation for acid production.

The research results also indicate that the TS content significantly impacts VFA production. Groups with higher solid content generate more VFAs during hydrolysis, and as cultivation time extends, VFAs accumulate. This suggests that organic acids are produced but not fully consumed during methanogenesis. Its acid-production efficiency is comparable to that of the inoculated system, subverting the traditional perception that “exogenous inoculation dominates acid production” and providing new ideas for the development of low-energy-consumption AD processes. However, high VFAs also inhibited the microbiome; previous research also reported that VFAs even inhibited the acidogenic bacteria in the order of valeric propionic acid > butyric acid > isobutyric acid > acetic acid during pig manure digestion [25].

### 3.4. Organic Matter Transformation via the Four Classical AD Stages

**Figure 4:**
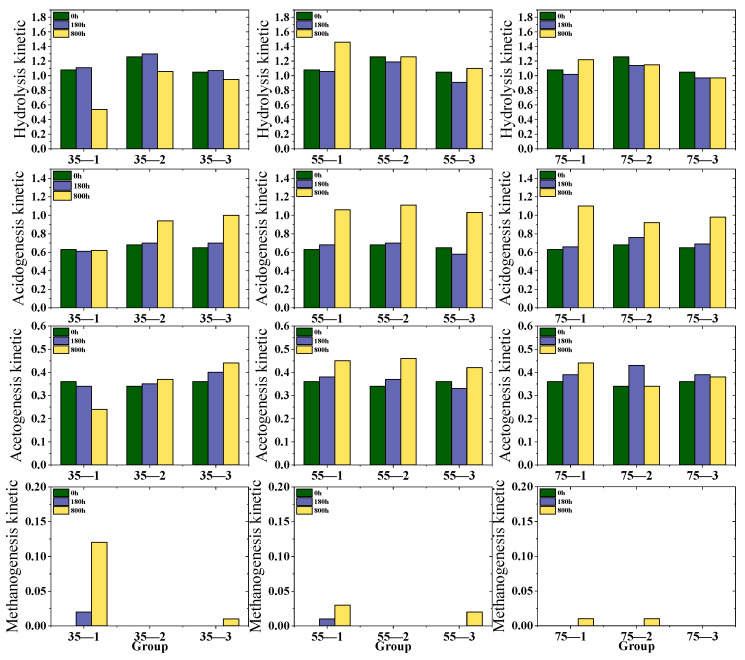
Effects of temperature and TS on hydrolysis, acidification, acetogenesis, and methanogenesis as well as Kinetic Parameters of methanogenesis.

Figure 4 depicts the numerical correlations among different experimental groups across the four key stages of anaerobic digestion: hydrolysis, acidification, acetogenesis, and methanogenesis. Notably, during the hydrolysis stage, certain samples exhibit a hydrolysis efficiency exceeding 100%, likely due to the inherent inaccuracies in the solid COD testing method. It is evident that the hydrolysis efficiency at 55 °C is comparatively high. In terms of acidification, 55 °C outperforms 35 °C and is significantly higher than 75 °C. Regarding acetogenesis, at 35 °C (mesophilic conditions), the hydrolysis efficiency hovers around 40%, while at 55 °C, it can surpass 40% over time. The trends at 75 °C and 55 °C show some resemblance.

During methanogenesis, the 35-1 sample at 35 °C demonstrates a relatively high efficiency, with a peak exceeding 12%. Conversely, at 55 °C and 75 °C, the methanogenesis efficiencies are substantially lower, with the maximum at 55 °C merely around 3% and that at 75 °C being even less. The composition of VFAs generated (Figure 3) during acidification and acetogenesis is crucial for AD and microbial communities under high-ammonia conditions [26]. A more in-depth exploration of the relationship between temperature and the kinetic laws of high-solid-content anaerobic digestion is still needed, which will enable more precise process control.

### 3.5. EPS Changes Response to the Key Inhibitors of FA and FVFA

**Figure 5:**
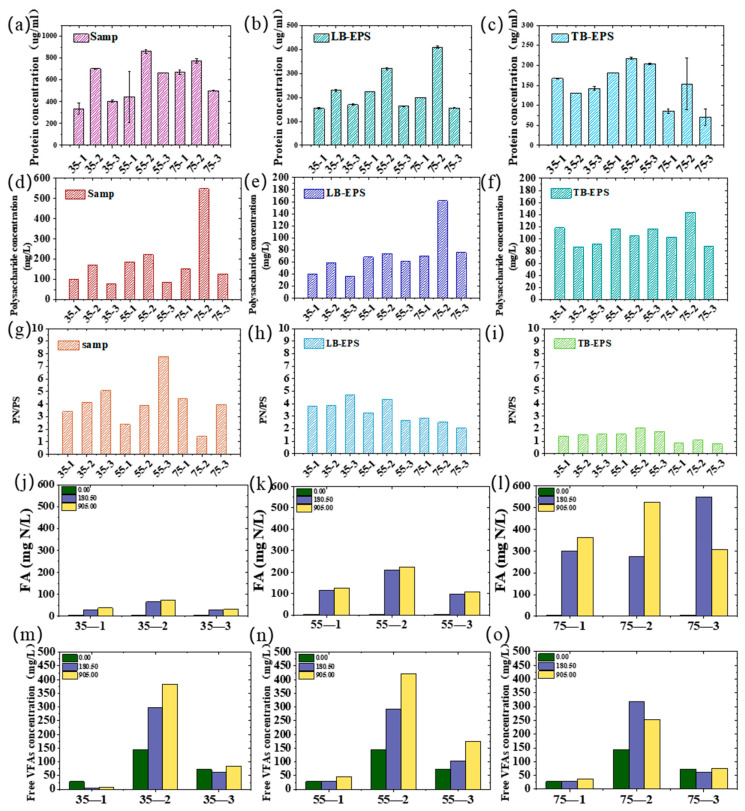
Protein and, polysnacharide andPN/PS changes of indivaral Samp (**a**,**d**,**g**), LB-EPS (**b**,**e**,**h**), TB-EPS (**c**,**f**,**i**) and key inhibitory factors of FA (**j**–**l**) and FVFA (**m**–**o**) at 35 °C, 55 °C and 75 °C following time.

The concentration of FA and FVFA are pH, concentration, and temperature-dependent. In all tests, the pH appears stable due to the buffering effect of ammonia (Table S2). Figure 5 illustrates EPS changes and key inhibitory factors of FA and FVFA variation. The three leys of EPS increased following temperature (Figure 5a–f), as well as a positive correlation to with high FA (Figure 5j–l), which may be due to microorganisms secreting a large amount of EPS especially proteins to alleviate FA inhibition (Figure 5g–i). A lower PS/PN ratio indicates a higher protective capability. Extracellular proteins, with their strong hydrophobic properties, can form an armor-like layer to protect cells [7]. The composition of EPS with EEM of each lay is illustrated in Figure S6. The toxic effect of high ammonia concentrations in CM on anaerobic bacteria poses a significant challenge to anaerobic digestion. A high ammonia concentration (TAN) of 7000 mg/L(Figure S4) and a high FA of 600 mg/L were calculated based on Equation (6). However, posited to the report [27] that FA can steer the anaerobic digestion process towards biohydrogen production in our steady, even with high VFA accumulation. The accumulation of ammonia initially suppresses methanotrophs, preventing the timely digestion of organic acids and consequently giving rise to a time-series cumulative effect. High concentrations of volatile fatty acids (VFA) exert an adverse effect due to the free VFA, which can transfer into cells freely inhibiting the functional metabolic activity of microorganisms within the community (Figure S5 individual FVFA).

The ammonia inhibition level in the AD process of manure ranges from 2.6 to 8.0 g/L [28,29,30]. Generally, high ammonia levels could lead to a change in intracellular pH, an increment in maintenance energy requirement, depletion of intracellular potassium, and inhibition of specific enzyme reactions [31,32]. During the process of cell repair in which microorganisms resist ammonia inhibition requires the cells to consume additional energy in order to pump potassium ions out of the cells, resulting in potassium depletion. The continuous accumulation of VFA creates an acidic environment, which is favorable for hydrogen-producing bacteria. It has been reported that the inhibitory threshold of volatile fatty acids is 6.0 g/L [33]. Since propionate is prone to accumulation and difficult to degrade, it is the cause of acidification in anaerobic digestion systems.

However, owing to the heterogeneity of the substrates, cases of metastable fermentation under high concentrations of ammonia nitrogen have also been reported frequently. In our previous report, the process could exhibit recovery behavior even when exposed to total ammonia nitrogen as high as 16,000 mg/L [8]. Notably, in the process of extremely high-solid digestion, certain other organic substances, particularly polar pollutants, present a significant threat to the integrity of bacterial membranes. Specifically, they disrupt the delicate ion gradient across the membranes. This disruption subsequently leads to membrane swelling and an increase in permeability. Ultimately, these adverse effects culminate in cell lysis. It is widely recognized that the anaerobic process is highly sensitive to a diverse range of organic compounds, including but not limited to halogenated aliphatic alkanes and alcohols. These organic compounds like aromatic chemicals and antibiotics can exert inhibitory effects on anaerobic digestion by interfering with the key enzymatic reactions or metabolic pathways that are integral to the anaerobic process [34]. Therefore, conducting more in-depth research on the impact of emerging pollutants collected with ammonia in CM with high-solid content on anaerobic fermentation is a direction that requires continuous exploration.

### 3.6. Microbial Community Succession and Self-Assembly

**Figure 6:**
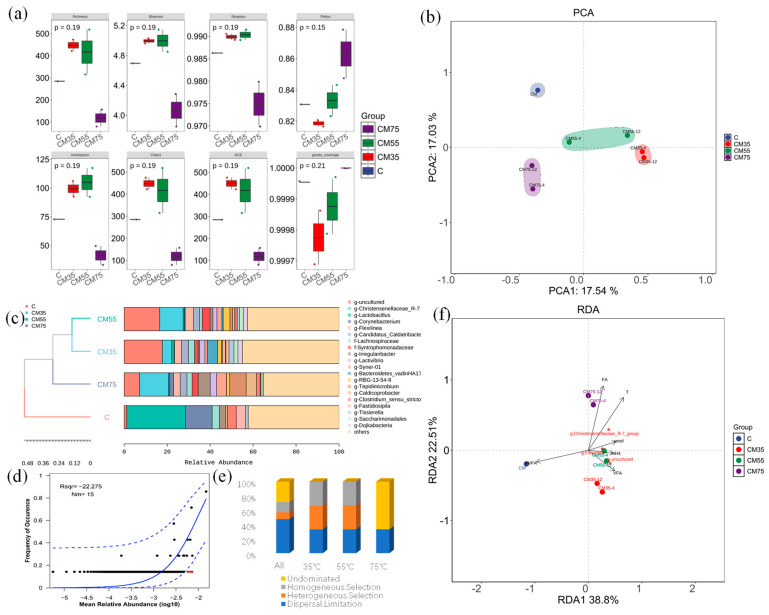
Alpha and Beta diversity (**a**,**b**) of the community, structure (**c**), Neutral model for Microbial Assembly (Dashed lines represent 95% confidence intervals around the neutral community model prediction. OTUs that occurred less frequently than predicted is displayed in red) (**d**), Null Model (Deterministic Processes) for Microbial Assembly Mechanisms (**e**) and the RDA analysis of the tests (**f**). Note: * represents the microbiota in (**f**).

The relative abundance of the dominant phylum of the microbial community from each sample is shown in Figure 6 and Figure S7 at different taxonomic levels. Overall, more than 31 phyla and more than 350 genera were detected among the samples. The phyla Firmicutes, Bacteroidota, Chloroflexi, and Actinobacteriota were the most dominant bacteria in all samples, accounting for more than 85% of the total community (Figure S8). Firmicutes maintained a relatively high abundance across all samples, demonstrating their robust adaptability to various environmental conditions. Actinobacteriota accounted for 11% in the CK sample, but its proportion decreased to 5% in 35 °C and 55 °C samples and further dropped to 2% in the high-temperature treatment group, suggesting high-temperature inhibition on its growth and survival. Bacteroidota showed 10% abundance at 35 °C, yet varied significantly and was below 9% in other samples, likely due to differences in substrate availability and metabolic pathways as it is involved in complex organic matter degradation. Proteobacteria’s abundance surged to 11% and 20% in high-temperature samples 75-1 and 75-2, respectively, contrasting with less than 1% at 35 °C. Chloroflexi, with 12% abundance in both 35 °C and 55 °C samples, is presumed to play a vital role in the relevant ecological processes at these temperatures. The comparison of the alpha diversity indicators of the samples showed that high temperature significantly decreased the diversity (decreased to one-fifth of the original value) [35], and the optimized conditions were conducive to the metabolism, driving a high diversity (Figure 6a). Interestingly, in contrast to most reports suggesting a high diversity of CM gut microbiota [36], our study revealed that in the absence of inoculum, only three phyla were predominantly concentrated (Bacteroidota, Firmicutes, and Actinobacteriota) accounting for over 99% of the total bacterial composition.

Nevertheless, a high level of diversity was observed at the genus level, indicating a high abundance of metabolic pathways. Significant differences in Beta diversity among the samples, with temperature as the main dividing line, clustered the samples, reflecting significant differences in the community structure (Figure 6b). These variations in genus-level abundances across samples suggest that environmental factors of temperature and TS play a crucial role in shaping the microbial community structure, influencing the distribution and abundance of different microbial genera.

The microbial self-assembly mechanism was analyzed using both the null model and the neutral community model. Overall, judging from the neutral community model (low Rsqr and Nm), the assembly of communities under different temperature conditions is less influenced by stochastic processes (Figure 6d). As detailed in Table S3, we calculated the phylogenetic null model (βNTI) and the taxonomic unit null model (RCbray). Based on the βNTI values, the succession at 35 °C and 55 °C was primarily driven by deterministic processes (e.g., selection), including dispersal limitation, heterogeneous selection, and homogeneous selection, which may contribute to the high ammonia generated from high TS (Figure S4). In contrast, succession at 75 °C, specifically in sample 75-1 to CM, was predominantly characterized by dispersal limitation, while other samples of 75 °C were driven by undominated processes (Figure 6d), this undominated process mainly caused by the high-temperature sterilization effect [37]; furthermore, high temperature favors the selection of more capable bacteria and methanogens in the high TS digestion [38].

Among identified bacterial genera, *Lactobacillus* (>25%, this is related to the probiotics added during the feeding process) was the predominant genus in the uninoculated sample with only intestinal parasitic bacteria, accounting for 25.2%, followed by *Corynebacterium* (11.3%), *Weissella* (4%), *Bacteroides* (4%), *g__Christensenellaceae_R-7_group* (4%), *g__Bacteroidetes_vadinHA17* (4%). Some intestinal parasitic bacteria can also secrete a variety of antimicrobial peptides, enzymes, and short-chain fatty acids, thereby improving the intestinal environment and maintaining the health of the host [36].

For hydrolysis and acidogenesis, Lactobacillus abundance decreased with rising temperature, and it was only detected at <1% in high TS conditions at 55 °C and 75 °C. This also proves that *Lactobacillus* are extremely likely to lose their dominant position in high organic conditions [39] and disappear functionally once they leave the intestinal environment [40]. In contrast, Christensenellaceae_R-7 emerged as a thermotolerant keystone taxon, accounting for 3% in 35-1 and 5% in 35-2 at 35 °C, 12% in 55-1 and 9% in 55-2 at 55 °C, and 8% in 75-1 and 14% in 75-2 at 75 °C. This group plays a vital role in the decomposition and transformation of organic carbon, especially in polysaccharide degradation. Concurrently, Candidatus_Caldatribacterium is one of the dominant bacterial communities in the anaerobic digestion system. Its proportion remains relatively stable at around 3% across different temperatures. It plays a crucial role in acid production, participates in the syntrophic oxidation of acetate, and has electron-transfer potential. It is closely related to nitrogen and phosphorus nutrient removal, can tolerate chemical stress, and shows strong resistance and ecological adaptability. In addition, this bacterium participates in the reaction of reducing nitrite to ammonia (DNRA) during the denitrification process [41]. The Bacteroidetes_vadinHA17 genus exhibited temperature-dependent abundance, peaking at 35 °C (4–5% in duplicates) but declining sharply (< 1.1%) under thermophilic conditions (55–75 °C). This genus contributes to protein/amino acid metabolism and acidogenesis, critical for methanogenesis during polycyclic aromatic hydrocarbon degradation [42]. Notably, hydrolytic bacteria Tepidimicrobium dominated at 75 °C (16% in 75-1), displaying broad metabolic versatility (carbohydrate/protein utilization) and acid/hydrogen production via the EMP pathway [43]. Its symbiotic relationship with Methanothermobacter enables concurrent hydrogen and methane generation, highlighting potential for bioenergy optimization. Caldicoprobacter, which was prevalent at 55 °C (accounting for 9–12% of the community), exhibited thermophilic (>50 °C) capabilities for degrading polysaccharides and proteins, producing acetic and butyric acids, CO_2_, and hydrogen—key substrates for hydrogenotrophic methanogens. Proteiniphilum, a mesophilic bacterium prevalent at 35 °C (1–4%) and less than 1% at 55 °C, could efficiently degrade proteins into amino acids and volatile fatty acids (VFAs). The novel filamentous anaerobe *Flexilinea flocculi* (abundance 1–4% of the samples) produced multiple acids and hydrogen, with its growth enhanced through co-culturing with hydrogenotrophic methanogens.

For methanogenesis, twelve methanogenic genera were identified (Figure S8), dominated by hydrogenotrophic *Methanobacterium* among all samples (35 °C: >37% low-solid, >32% high-solid; 55 °C: >0.3% low-solid, >23% high-solid; 75 °C: >16% low-solid, >46% high-solid). Acetoclastic *Methanosaeta* showed temperature-dependent shifts, peaking at 35 °C (28.7–45.1%) and 55 °C (43.4% in low-solid, 18% in high-solid), but not detected in the ultra-high-solid content process at 75 °C, confirming that the *Methanosaeta* is sensitive for the temperature and inhibited under high TS and high FA (Figure 3 and Figure 4). Methylotrophic *g__Candidatus_Methanofastidiosum* occupied 1% in an extremely high solid of 55 °C, which is an autotrophic microorganism that can utilize carbon dioxide as the sole carbon source [44], reflecting the high metabolic diversity of the community. *Methanolinea* has metabolic synergistic effects with other important methanogenic archaea, such as *Methanoculleus* and *Methanobacterium*, jointly maintaining the methanogenesis process in anaerobic digestion systems (Figure S8).

Thus, temperature stratification drives microbial consortia specialization, with distinct acidogenic/hydrogen-producing genera (*Bacteroidetes_vadinHA*17, *Tepidimicrobium*) favoring mesophilic conditions. The cross-feeding symbiosis between fermentative bacteria (*Caldicoprobacter*, *Flexilinea*) and hydrogenotrophic methanogens enhances carbon flux toward methane. Thermophilic adaptation and high TS reconstructed methanogen dominance, favoring hydrogenotrophs (*Methanobacterium*) over acetotrophs (*Methanosaeta*) for CH_4_ generation.

### 3.7. Microbial Community Metabolism Functional Prediction

**Figure 7:**
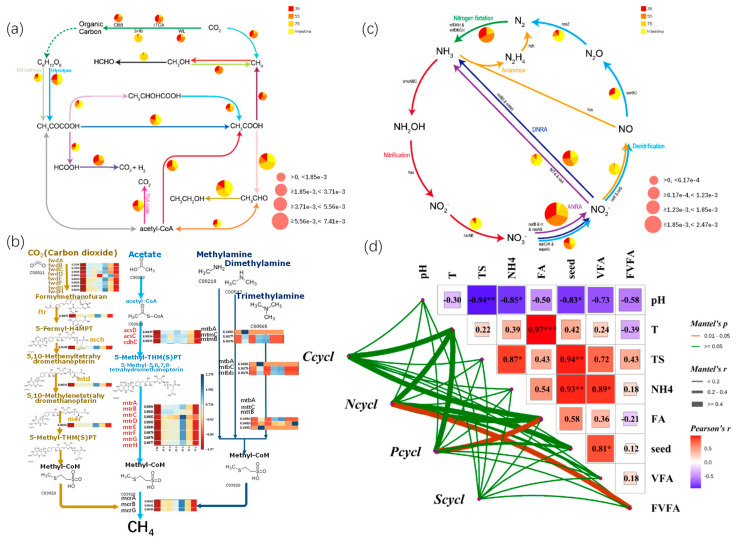
Proposed carbon cycle metabolic pathways at different temperatures (**a**) Three major methanogenic pathways (**b**), nitrogen cycle pathway (**c**) Mental analysis of KO abundance related to C, N, P, and S cycle (**d**). * represents *p* < 0.05, ** represents *p* < 0.01, *** represents *p* < 0.001.

A meticulous KEGG Orthology (KO) analysis was carried out to comprehensively explore the essential characteristics of the microbial community, as clearly presented in Figure 7. Through accurate functional prediction by Tax4Fun2 with a highly known fraction (Figure S9), the analysis clearly showed significant differences in KOs between mesophilic and thermophilic bacteria; similar results were also reported in previous work [8]. This statistical significance validates the authenticity and importance of these findings. The relative abundances of KO were systematically collated and tabulated in Tables S4–S6. Notably, both intestinal microbiomes and those acclimated to inoculation at a high temperature of 75 °C exhibited an extremely strong sulfate-reduction function (Figure S10). This phenomenon implies a remarkable enhancement in community succession and metabolic functional redundancy of microbial communities. The metabolic pathways for hydrogen sulfide production via sulfate reduction were more diverse at 35 °C than at 55 °C. Functionally, when focusing on the carbon cycle (Figure 7a) provided a detailed visualization of microbial gene abundances. The ASPB gene, with the highest abundance and a close connection to the carbon cycle in all samples, was identified as crucial for organic carbon oxidation. The LDHG gene, involved in anaerobic lactate metabolism, ranked second in abundance, and the ACK gene, functioning in the acidification process, was third. In the 35-1 sample at 35 °C, the ASPB gene had the highest abundance and dominated the carbon cycle and acid oxidation in organic metabolism. The FDOG gene, participating in the conversion of acetic acid to acetyl-CoA, ranked third, and the FDHAG gene, a key gene for formic acid oxidation, ranked fourth. The 35-2, 55-1, and 55-2 samples showed the same pattern. Compared with 35 °C, at 50 °C, the abundances of the ACDA gene (related to acidification) and the FBOH gene (involved in thyroid oxidation) increased significantly, and the abundance of the CDHE gene involved in the Wood-Ljungdahl pathway also rose markedly. These findings provided important insights into a deeper understanding of microbial metabolism and environmental adaptability.

In the methanogenesis pathways (Acetotrophic, Hydrogenotrophic, Methylotrophic methanogenesis) (Figure 7a,c), a distinct temperature-related pattern emerged, with 55 °C showing the highest activity for all three methanogenesis types. In the distribution of functional genes for hydrogenotrophic pathway to methane, 55 °C had the highest proportion, followed by 35 °C. In the processes of lactate conversion to methane and methane production by acetotrophic methanogens, the proportions at 35 °C and 55 °C were relatively high, while those at 75 °C and for gut microbiota were nearly zero. In the metabolic pathway of methylotrophic methanol conversion to methane, the proportion at 55 °C exceeded 75%, and that at 35 °C was approximately 35%. Among them, the 55-2 sample (55 °C) had the highest methanogenesis pathway activity, followed by the 35-1 sample (35 °C).

In the nitrogen cycle (Figure 7b), significant differences were observed among the three groups of microorganisms. During the conversion of nitrate to nitrite, the gene abundance proportion was highest at 35 °C, followed by 55 °C, then 75 °C and gut microbiota. In the process of dissimilatory nitrate reduction to ammonium (DNRA), the gene abundance proportions of the four groups of microorganisms were similar. Meanwhile, nitrogen-cycle genes were only significantly correlated with FVFA, and phosphorus-cycle-related genes in the microbial community were significantly positively correlated with free ammonia (Figure 7d). These significant relationships were highly consistent with the data patterns of hydrolysis-acidification, acetification (Figure 4), and FA, FVFA mentioned above (Figure 5), providing crucial evidence for a deeper understanding of the metabolic mechanisms and ecological functions of microorganisms under different environmental conditions. These findings offer deep-seated insights into microbial metabolism and environmental adaptability, which is fundamental for further research in this domain.

### 3.8. Environmental Implications of the Present Work

In response to the engineering challenge of the unknown anaerobic treatment efficiency of high-solid-content manure under extreme piling conditions, this study focuses on the efficiency attenuation mechanism of the “hydrolysis-acidification-acetification-methanation” metabolic chain. By establishing a dual-factor experimental system of gradient temperatures (4 °C, 35 °C, 55 °C, 75 °C) and inoculation regulation, it systematically reveals the metabolic laws of intestinal microorganisms and inoculated functional flora in the high TS system. For the first time, this study quantitatively analyzes the metabolic overload phenomenon of acid-producing bacteria under 55 °C high-temperature stress (12 g/L) and the thermal inactivation boundary of methanogens. This fills the research gap in the inhibition links between hydrolysis, acidification, acetification, and methanation during the anaerobic conversion of extremely high TS conditions.

## 4. Conclusions

This research focuses on the anaerobic digestion of high-solid chicken manure, exploring the impacts of temperature and total solid content (TS) on the process. Temperature plays a vital role in methane production. At 35 °C, the system attains the highest methane production efficiency, with a yield of 68 mL/gVS. In contrast, high temperatures like 55 °C and 75 °C, along with high TS levels, lead to an ammonia nitrogen increase beyond 6000 mg/L, resulting in a 98.6% decline in methane yield. For instance, at 55 °C with 12% TS, high concentrations of free ammonia (FA > 600 mg/L) and free volatile fatty acid (FVFA > 450 mg/L) significantly suppress CH_4_ yield drops to 0.92 mL/gVS. Additionally, high TS and temperature cause the accumulation of volatile fatty acids (VFAs) up to 12 g/L. Intriguingly, the chicken intestinal microbiota, even without sludge inoculation, shows strong hydrolytic and acidogenic capabilities with VFA accumulation reaching a maximum of 11 g/L. Although high temperatures reduce microbial diversity, heat-resistant bacteria such as *Tepidimicrobium* and *Caldicoprobacter* become dominant, which drives the carbon-nitrogen cycle and the mineralization of organic matter. The cross-feeding symbiosis between fermentative bacteria (*Caldicoprobacter*, *Bacteroidetes*, *Tepidimicrobium*) and hydrogenotrophic *Methanobacterium* significantly enhances methane production at 35 °C. Overall, this study accurately analyzes the effects of temperature and inoculation factors on the acidification efficiency of high-solid chicken manure digestion. It provides essential scientific support for optimizing the resource utilization of chicken manure waste.

## Data Availability

The original contributions presented in this study are included in the article/Supplementary Material. Further inquiries can be directed to the corresponding author.

## Supplementary Materials

The following supporting information can be downloaded at https://www.mdpi.com/article/10.3390/microorganisms13040724/s1, Figure S1. 3D-EEM of DOM variation during operational process. Figure S2. Correlation of the four phases of each test over time. Figure S3. The COD distribution of the tests. Figure S4. TAN concentration of each testes. Figure S5. FVFA calculation of different temperature tests. Figure S6. 3D-EEM analysis of each kinds of EPS. Figure S7. Heatmap of all phyla (a) and the top 50 genera of the microbial community. Figure S8. Heatmap of Methanogens in genus level and the correlation. Figure S9. The accuracy of functional prediction by Tax4Fun for each sample. Figure S10. Abundance of sulfur cycle-related genes for community function prediction. This figure was drawn using an online website. Our gratitude goes to this website: https://www.cloudtutu.com. Table S1. Experimental design and key parameters of biotransformation at different temperatures. Table S2. pH variation during the operation time. Table S3. The microbial self-assembly mechanism. Table S4. The variation in the number of key KOs related to the nitrogen cycle. Table S5. The variation in the number of key KOs related to the phosphorus cycle. Table S6. The variation in the number of key KOs related to the carbon cycle.
