# Supplementary material for: Anaerobic Digestion of High-Solid Chicken Manure (CM) at Different Temperature: Intestinal Microbiome Efficiency, Inhibition, and Microbial Community Evolution
Source: Microorganisms. 2025 Mar 24;13(4):724. doi: 10.3390/microorganisms13040724 (PMC12029704; doi:10.3390/microorganisms13040724)
Supplement: Supplementary file 1 [file microorganisms-13-00724-s001.zip › microorganisms-3519732-supplementary.pdf]

Supplementary material for

Anaerobic Digestion of High-Solid Chicken Manure (CM) at Different Temperature: Intestinal Microbiome Efficiency, Inhibition, and Microbial Community Evolution

Xujing Chen [1], Qigui Niu [1,2,3,*], Jingyi Li [1], Zijing Zhou [1], Yue Wu [1], Guixue Song [4] and Rutao Liu [1,2]

1 China-America CRC for Environment & Health, School of Environmental Science and Engineering, Shandong University, Qingdao 266237, China; 202222210066@mail.sdu.edu.cn (X.C.); 202012949@mail.sdu.edu.cn (J.L.); 202200210019@mail.sdu.edu.cn (Z.Z.); 202332960@mail.sdu.edu.cn (Y.W.); rutaoliu@sdu.edu.cn (R.L.)

2 Shandong Provincial Key Laboratory of Water Pollution Control and Resource Reuse, Shandong University, Qingdao 266237, China

3 Shenzhen Research Institute, Shandong University, A301 Virtual University Park in South District, Shenzhen 518000, China

4 Institute of Marine Science and Technology, Shandong University, Qingdao 266237, China; songgx@sdu.edu.cn

* Correspondence: niuqg@sdu.edu.cn

Table S1. Experimental design and key parameters of biotransformation at different temperatures

T (℃)	Tests	Sludge (g)	CM (g)	volume (mL)	liquid (mL)	TS	VS (g)	Biogas （ml）	Rate (mL/gVS)	CH_4 （ml）	Rate (mL/gVS)	CO_2 （ml）	mL/gVS	H_2 （ml）	Rate (mL/gVS)
4℃	4—1	6	20	80	54	5.07%	2.48	0.00	0.00	0.00	0.00	0.00	0.00	0.00	0.00
	4—2	15	47	80	18	12.05%	5.83	0.00	0.00	0.00	0.00	0.00	0.00	0.00	0.00
	4—3	0	20	80	60	4.21%	2.48	0.00	0.00	0.00	0.00	0.00	0.00	0.00	0.00
	4—4	0	47	80	33	9.90%	5.83	0.00	0.00	0.00	0.00	0.00	0.00	0.00	0.00
35℃	35—1	6	20	80	54	5.07%	2.48	385.40	155.39	169.18	68.21	155.0	62.50	0.00	0.00
	35—2	15	47	80	18	12.05%	5.83	86.50	14.84	5.36	0.92	27.00	4.63	1.26	0.22
	35—3	0	20	80	60	4.21%	2.48	30.30	12.22	11.61	4.68	15.00	6.05	1.65	0.66
	35—4	0	47	80	33	9.90%	5.83	36.80	6.31	0.90	0.15	3.37	0.58	1.26	0.22
55℃	55—1	6	20	80	54	5.07%	2.48	80.40	32.42	2.51	1.01	18.70	7.54	0.14	0.06
	55—2	15	47	80	18	12.05%	5.83	75.00	12.87	0.66	0.11	9.12	1.56	2.04	0.35
	55—3	0	20	80	60	4.21%	2.48	45.50	18.35	0.00	0.00	10.00	4.03	7.05	2.84
	55—4	0	47	80	33	9.90%	5.83	48.00	8.24	0.00	0.00	7.50	1.29	0.01	0.00
75℃	75—1	6	20	80	54	5.07%	2.48	40.50	16.33	0.67	0.27	10.30	4.15	0.06	0.02
	75—2	15	47	80	18	12.05%	5.83	55.40	9.51	0.19	0.03	7.86	1.35	0.00	0.00
	75—3	0	20	80	60	4.21%	2.48	36.20	14.60	0.00	0.00	4.50	1.81	0.00	0.00
	75—4	0	47	80	33	9.90%	5.83	66.20	11.36	0.00	0.00	9.10	1.56	0.00	0.00

Table S2 pH variation during the operation time

pH	35—1	35—2	35—3	55—1	55—2	55—3	75—1	75—2	75—3
0	7.80	7.50	7.40	7.80	7.50	7.40	7.80	7.50	7.40
180	8.50	7.20	7.50	7.80	7.20	7.20	7.80	7.20	7.50
800	8.50	7.20	7.50	7.80	7.20	7.20	7.80	7.20	7.50

Table S3 The microbial self-assembly mechanism

Station1	Station2	bMNTD	BC	bNTI	RC	Self-assembly Process
CM35-12	CM	0.19	1.00	1.21	1.00	Dispersal.Limitation
CM35-4	CM	0.25	1.00	3.57	1.00	Heterogeneous.Selection
CM35-12	CM35-4	0.08	1.00	-2.90	1.00	Homogeneous.Selection
CM55-12	CM	0.24	1.00	4.39	1.00	Heterogeneous.Selection
CM55-4	CM	0.18	0.98	0.88	1.00	Dispersal.Limitation
CM55-12	CM55-4	0.10	1.00	-2.08	1.00	Homogeneous.Selection
CM75-12	CM	0.21	0.95	0.01	-0.30	Undominated
CM75-4	CM	0.16	0.99	-0.68	0.98	Dispersal.Limitation
CM75-12	CM75-4	0.16	0.99	-1.33	-0.08	Undominated

Table S4 The variation in the number of key KOs related to the nitrogen cycle

KO	gene_name	function	Category	Intestinal	35℃-1	35℃-2	55℃-1	55℃-2	75℃-1	75℃-2
K02591	nifK	N2 fixation	step1_Nitrogen_fixation	2.14E-06	0.000209	0.000128	0.000103	0.000197	0.000123	0.000131
K15876	nrfH	Nitrite reduction to ammonia	step8_Nitrite_ammonification	2.85E-05	0	1.42E-06	1.04E-07	0	5.62E-07	0
K04015	nrfD	Nitrite reduction to ammonia	step8_Nitrite_ammonification	2.97E-06	0	0	0	0	0	0
K07218	nosD	Nitrous oxide reduction	step7_Nitrous_oxide_reduction	1.52E-05	0.000505	0.000592	0.00061	0.000454	0.00041	3.73E-05
K02568	napB	Nitrate reduction	step4_Nitrate_reduction	6.23E-06	4.60E-07	3.80E-07	0	0	0	0
K02567	napA	Nitrate reduction	step4_Nitrate_reduction	9.77E-06	0.000179	0.000274	0.00032	0.000514	0.00024	4.49E-05
K02588	nifH	N2 fixation	step1_Nitrogen_fixation	2.38E-05	0.000477	0.000315	0.000551	0.000728	0.00025	0.00017
K02586	nifD	N2 fixation	step1_Nitrogen_fixation	2.14E-06	0.000254	0.000167	0.000181	0.000374	0.000101	0.000157
K00376	nosZ	Nitrous oxide reduction	step7_Nitrous_oxide_reduction	3.77E-06	8.42E-07	3.80E-07	0	1.71E-06	0	0
K00371	narH	Nitrate reduction	step4_Nitrate_reduction	0.000159	2.24E-05	2.03E-05	7.07E-06	2.81E-06	5.89E-06	3.44E-05
K00370	narG	Nitrate reduction	step4_Nitrate_reduction	0.000161	2.24E-05	2.00E-05	8.01E-06	3.20E-06	2.23E-05	3.47E-05
K02305	norC	Nitric oxide reduction	step6_Nitric_oxide_reduction	6.95E-06	8.42E-07	5.32E-07	0	1.71E-06	0	0
K00531	anfG	N2 fixation	step1_Nitrogen_fixation	0	1.49E-05	0	0	0	0	0
K03385	nrfA	Nitrite reduction to ammonia	step8_Nitrite_ammonification	4.27E-05	0	1.42E-06	1.04E-07	0	5.62E-07	0
K15864	nirS	Nitrite reduction	step5_Nitrite_reduction	4.28E-06	4.60E-07	6.08E-07	0	1.16E-06	0	0
K04561	norB	Nitric oxide reduction	step6_Nitric_oxide_reduction	3.99E-05	1.90E-05	2.02E-05	0	3.34E-06	0	2.48E-06
K00368	nirK	Nitrite reduction	step5_Nitrite_reduction	9.61E-05	3.83E-07	1.67E-06	5.51E-06	6.99E-07	1.40E-05	0.000212
K00362	nirB	Nitrite reduction to ammonia	step8_Nitrite_ammonification	0.000388	0.000538	0.000655	0.000551	0.000939	0.000412	0.000406
K00363	nirD	Nitrite reduction to ammonia	step8_Nitrite_ammonification	2.22E-05	2.05E-05	2.05E-05	2.21E-05	1.09E-05	0.000154	9.43E-05

Table S5 The variation in the number of key KOs related to the phosphorus cycle

KO	gene_nam	function	Category	Intestinal	12785	12816	20090	20121	27395	27426
K01126	ugpQ	glycerophosphoryl diester phosp	transports	0.000824	0.00069	0.000686	0.000549	0.000451	0.000508	0.00036
K00873	PK	pyruvate kinase	Polyphosphate degradation	0.0005	0.000298	0.000291	0.000274	0.000116	0.000219	0.000241
K09474	phoN	acid phosphatase (class A)	Organic P mineralization	0.000028	4.37E-06	1.9E-06	3.86E-05	0.000031	0.000435	2.89E-05
K00858	ppnK	NAD+ kinase	Polyphosphate degradation	0.000489	0.000539	0.000497	0.000611	0.000978	0.000431	0.000256
K00117	gcd	quinoprotein glucose dehydroge	inorganic P solubilization	1.78E-05	1.97E-07	7.06E-06	1.57E-06	6.05E-06	0.000109	0.000321
K02041	phnC	phosphonate transport system, A	transports	0.001334	0.000478	0.000638	0.000767	0.000593	0.000825	0.001532
K02040	pstS	phosphate transport system, per	transports	0.000606	0.000909	0.000997	0.001059	0.000907	0.000919	0.000438
K02043	phnF	C-P lyase subunit, GntR family tra	transports	4.03E-06	3.83E-07	1.75E-06	5.22E-07	9.31E-07	1.27E-05	0.000137
K02042	phnE	phosphonate transport system, r	transports	0.000461	0.000022	0.000047	1.21E-05	5.74E-06	9.25E-05	0.000191
K06162	phnM	C-P lyase subunit, alpha-D-ribos	Organic P mineralization	1.06E-05	3.83E-07	2.58E-06	2.52E-06	1.13E-05	5.38E-05	0.00011
K06163	phnJ	C-P lyase subunit, alpha-D-ribos	Organic P mineralization	5.66E-06	0	7.59E-07	5.22E-07	3.88E-07	1.27E-05	6.81E-05
K06166	phnG	C-P lyase subunit, alpha-D-ribos	Organic P mineralization	4.89E-06	0	7.59E-07	5.22E-07	3.88E-07	1.27E-05	6.81E-05
K06167	phnP	C-P lyase subunit, phosphoribosy	Organic P mineralization	6.71E-05	0.000262	0.000357	0.000351	0.000334	0.000303	0.000153
K06164	phnI	C-P lyase subunit, alpha-D-ribos	Organic P mineralization	5.66E-06	0	7.59E-07	5.22E-07	3.88E-07	1.27E-05	6.81E-05
K06165	phnH	C-P lyase subunit, alpha-D-ribos	Organic P mineralization	5.66E-06	0	7.59E-07	5.22E-07	3.88E-07	1.27E-05	6.81E-05
K01077	phoA	alkaline phosphatase	Organic P mineralization	0.000352	0.000499	0.00052	0.000605	0.000301	0.000449	0.000034
K03787	surE	5'/3'-nucleotidase	Polyphosphate degradation	3.34E-05	0.000414	0.000436	0.000371	0.000388	0.000275	0.00019
K03788	aphA	acid phosphatase (class B)	Organic P mineralization	3.84E-06	0	0	5.22E-07	0	0	0
K07048	opd	phosphotriesterase	Organic P mineralization	8.84E-06	5.09E-05	8.79E-05	5.22E-07	2.05E-05	0	2.45E-06
K05781	phnK	C-P lyase subunit, alpha-D-ribos	transports	0.000143	0.000232	0.000241	0.000134	0.000038	1.79E-05	7.16E-05
K05780	phnL	C-P lyase subunit, alpha-D-ribos	Organic P mineralization	7.09E-05	5.14E-05	9.08E-05	1.27E-05	1.47E-05	0.000111	0.000232
K05306	phnX	phosphonoacetaldehyde hydrola	Organic P mineralization	0.000103	3.76E-06	4.32E-06	5.58E-06	4.26E-06	7.65E-05	0.000092
K00940	ndk	nucleoside-diphosphate kinase	Polyphosphate degradation	0.000272	0.000326	0.000386	0.000414	0.000459	0.000365	0.000198
K01093	appA	4-phytase	Organic P mineralization	0.000018	2.18E-06	9.11E-07	1.96E-05	9.08E-06	9.35E-05	0
K01113	phoD	alkaline phosphatase	Organic P mineralization	9.92E-05	3.67E-05	4.17E-05	7.07E-06	1.12E-05	0.000185	0.000146
K01139	spoT	GTP diphosphokinase / guanosin	Polyphosphate degradation	0.000315	0.000114	0.00019	0.00016	6.07E-05	0.000196	0.000199
K05813	ugpB	Glycerol-3-phosphate transporte	transports	0.000158	0.000199	0.00038	0.000381	5.65E-05	0.000047	0.000217
K05816	ugpC	Glycerol-3-phosphate transporte	transports	0.001061	0.000348	0.000338	0.000485	0.000265	0.000547	0.002418
K05815	ugpE	Glycerol-3-phosphate transporte	transports	0.000209	0.000292	0.000563	0.00047	7.65E-05	5.12E-05	0.000215
K05814	ugpA	Glycerol-3-phosphate transporte	transports	0.000212	0.000167	0.00032	0.000341	0.000092	0.000063	0.000214
K03430	phnW	2-aminoethylphosphonate-pyru	Organic P mineralization	0.000157	7.71E-06	7.46E-06	4.11E-05	3.88E-05	4.99E-05	4.77E-06
K09994	phnO	C-P lyase subunit, aminoalkylpho	Organic P mineralization	2.9E-06	0	0	0	0	0	0
K01524	ppx	exopolyphosphatase / guanosine	inorganic P solubilization	0.000641	0.00013	5.05E-05	0.000248	0.00026	0.000371	0.000291
K19670	phnA	phosphonoacetate hydrolase	Organic P mineralization	0	0	0	0	0	1.27E-05	0.000041
K00886	ppgK	polyphosphate glucokinase	Polyphosphate degradation	0.000152	4.16E-06	7.59E-07	3.14E-05	1.55E-07	2.45E-05	8.02E-05
K02038	pstA	phosphate transport system, me	transports	0.000528	0.001001	0.001002	0.001005	0.001102	0.000832	0.000257
K02039	phoU	phoR/phoB inhibitor protein pho	regulatory	0.000424	0.000862	0.000778	0.000846	0.000819	0.000661	0.00029
K02036	pstB	phosphate transport system, ATP	transports	0.001238	0.001378	0.001467	0.001284	0.001306	0.000706	0.000805
K02037	pstC	phosphate transport system, me	transports	0.000515	0.000987	0.000993	0.000985	0.001056	0.000772	0.000261
K15986	ppaC	manganese-dependent inorgani	Polyphosphate synthesis	0.000282	0.000182	0.000228	0.000232	0.000314	0.000127	4.21E-05
K03306	pit	inorganic phosphate transporter	transports	0.000148	0.000315	0.000296	0.000373	0.000555	0.000435	0.000252
K05774	phnN	C-P lyase subunit, ribose 1,5-bisp	Organic P mineralization	9.71E-06	3.83E-07	1.37E-06	5.22E-07	9.31E-07	1.27E-05	0.000109
K02044	phnD		transports	0.000272	1.26E-05	2.67E-05	6.46E-06	1.58E-05	8.26E-05	0.000209
K01507	ppa	inorganic pyrophosphatase	inorganic P solubilization	0.000134	0.000305	0.000175	0.000193	0.000167	0.000243	0.000201
K00937	ppk1	Polyphosphate kinase	Polyphosphate synthesis	0.000159	8.51E-05	4.11E-05	0.000118	0.000133	0.000207	0.000155
K00951	relA	GTP pyrophosphokinase	Polyphosphate degradation	0.000491	0.000219	0.000196	0.000207	0.000123	0.000231	0.000295
K07657	phoB	two-component system, OmpR f	regulatory	0.001838	0.001566	0.001475	0.00125	0.000783	0.001244	0.000897
K07636	phoR	two-component system, OmpR f	regulatory	0.002492	0.002078	0.001839	0.001753	0.001178	0.001195	0.000928

Table S6 The variation in the number of key KOs related to the carbon cycle

KO	gene_nam	function	Category	Intestinal	35℃-1	35℃-2	55℃-1	55℃-2	75℃-1	75℃-2
K00194	cdhD	Wood Ljungdahl pathway	step2_Carbon_fixation	2.08E-05	0.000252	0.00019	0.00024	0.000241	0.000146	2.35E-05
K00197	cdhE	Wood Ljungdahl pathway	step2_Carbon_fixation	2.08E-05	0.000491	0.000466	0.000539	0.000453	0.000372	3.79E-05
K00198	cooS	Wood Ljungdahl pathway	step2_Carbon_fixation	4.23E-05	0.000319	0.000253	0.000291	0.000379	0.000123	3.68E-05
K05825	aminotran:	Aminotransferase class I and	step1_Organic_carbon_oxida	0.000392	0.000337	0.000435	0.000445	0.00062	0.000385	0.000605
K00830	serine-pyr	Serine-pyruvate aminotransf	step1_Organic_carbon_oxida	4.19E-05	0.000297	0.00047	0.00026	0.000348	0.0002	0.00023
K00831	phosphose	Phosphoserine aminotransfe	step1_Organic_carbon_oxida	0.00026	0.000153	4.27E-05	0.000113	0.000106	0.000194	0.000153
K00812	aspB	Metabolism of organic sulfur	step1_Organic_carbon_oxida	0.001039	0.00158	0.001483	0.001664	0.001676	0.001014	0.00093
K00817	histidinol-p	Histidinol-phosphate/aroma	step1_Organic_carbon_oxida	0.000489	0.000741	0.000607	0.000849	0.000989	0.000564	0.000425
K00819	ornithine/a	Ornithine/acetylornithine am	step1_Organic_carbon_oxida	5.31E-05	0.000312	0.00025	0.000324	0.00052	0.000199	0.000362
K00656	pflD	Pyruvate <=> acetyl-CoA +	step6_Fermentation	0.000365	0.000176	0.000102	9.17E-05	9.49E-05	9.13E-06	5.94E-05
K05989	alpha-L-rh	Hemicullulose debranching	step1_Organic_carbon_oxida	0.000125	2.18E-06	5.69E-06	5.59E-05	3.49E-05	0.000162	3.44E-07
K01183	chitiniase	Chitin degrading	step1_Organic_carbon_oxida	4.50E-05	0.000195	0.000133	0.000123	0.000115	4.71E-05	1.03E-05
K15230	aclA	Reverse TCA cycle	step2_Carbon_fixation	2.20E-06	0.000141	0.000159	0.000163	0.000123	9.57E-05	1.29E-05
K15231	aclB	Reverse TCA cycle	step2_Carbon_fixation	0	0.000141	0.000159	0.000163	0.000123	9.39E-05	1.24E-05
K04115	bcrD	Benzoyl-CoA reduction	step1_Organic_carbon_oxida	0	7.16E-05	0.000144	5.82E-05	1.02E-05	0	0
K04114	bcrA	Benzoyl-CoA reduction	step1_Organic_carbon_oxida	4.78E-05	7.53E-05	0.000144	6.31E-05	1.19E-05	0	0
K19668	cellobiosid	Cellulose degrading	step1_Organic_carbon_oxida	0	0.000103	0	1.09E-05	1.67E-05	0	0
K00401	mcrB	Methane production	step7_Methanogenesis	0	0.000228	0.000212	0.000276	0.000403	0.000206	4.59E-05
K22515	fdwB	Formate oxidation	step1_Organic_carbon_oxida	7.83E-07	3.88E-05	7.57E-05	8.47E-05	4.20E-05	1.78E-06	4.20E-05
K22516	fdhA	Formate oxidation	step1_Organic_carbon_oxida	2.34E-05	0.000866	0.001155	0.001088	0.001076	0.000823	0.000362
K03520	coxL	Aerobic CO oxidation	step1_Organic_carbon_oxida	9.03E-05	0.000216	0.000429	0.000142	2.96E-05	0.000198	0.00085
K01905	acdA	Acetogenesis	step6_Fermentation	2.29E-05	0.000472	0.000472	0.000583	0.000785	0.000284	0.000557
K01070	fghA	Formaldehyde oxidation	step1_Organic_carbon_oxida	4.64E-05	2.76E-06	4.13E-06	2.13E-05	1.33E-05	0.000196	0.000137
K00016	ldh	Lactate utilization	step6_Fermentation	0.000805	0.00012	0.000198	0.000294	0.000464	0.000107	0.00025
K00153	mycoS_de	Formaldehyde oxidation	step1_Organic_carbon_oxida	0.000153	6.07E-06	4.18E-06	2.47E-05	1.18E-05	0.000147	0.000186
K01214	isoamylase	Amylolytic enzymes	step1_Organic_carbon_oxida	0.000142	5.30E-05	3.31E-06	7.00E-05	1.83E-05	0.000209	0.000147
K01218	mannan er	Endohemicellulases	step1_Organic_carbon_oxida	1.32E-05	0.00026	8.53E-05	0.000393	0.000398	1.32E-05	2.06E-06
K00925	ack	Acetogenesis	step6_Fermentation	0.00073	0.000324	0.000377	0.000325	0.000145	4.27E-05	0.000163
K01895	acs	Acetate to acetyl-CoA	step6_Fermentation	0.000139	0.00105	0.001117	0.00143	0.001928	0.00165	0.001036
K01176	alpha-amy	Amylolytic enzymes	step1_Organic_carbon_oxida	0.000325	0.000279	0.000279	0.000388	0.000104	0.000153	1.63E-05
K01178	glucoamyl	Amylolytic enzymes	step1_Organic_carbon_oxida	5.80E-05	2.18E-06	9.87E-07	2.47E-05	1.18E-05	0.000156	0.000125
K01612	bsdC	Phenol => Benzoyl-CoA	step1_Organic_carbon_oxida	1.64E-06	1.82E-05	1.89E-05	5.22E-07	0	0	4.10E-05
K00399	mcrA	Methane production	step7_Methanogenesis	0	0.000228	0.000212	0.000276	0.000403	0.000206	4.59E-05
K03186	ubiX	Phenol => Benzoyl-CoA	step1_Organic_carbon_oxida	7.58E-05	0.000279	0.000302	0.000282	0.00041	0.000288	0.000119
K00826	branched-	Branched-chain amino acid a	step1_Organic_carbon_oxida	0.000364	0.000529	0.000503	0.000601	0.000621	0.000522	0.000428
K00823	4-aminobu	4-aminobutyrate aminotrans	step1_Organic_carbon_oxida	0.000324	0.000567	0.000554	0.000484	0.000521	0.000477	0.000533
K14028	mxaF	Methanol oxidation	step1_Organic_carbon_oxida	3.21E-06	0	2.43E-06	0	9.31E-07	1.27E-05	8.21E-05
K01811	alpha-D-x	Endohemicellulases	step1_Organic_carbon_oxida	0.00014	3.20E-05	4.09E-05	6.07E-05	3.35E-05	9.89E-05	2.64E-06
K03421	mcrC	Methane production	step7_Methanogenesis	0	0.000228	0.000212	0.000276	0.000403	0.000172	4.59E-05
K13954	acetaldehy	Acetaldehyde => Ethanol	step1_Organic_carbon_oxida	0.000345	0.000158	0.000242	0.000102	8.95E-05	9.81E-05	0.000149
K01192	beta-man	Other oligosaccharide degra	step1_Organic_carbon_oxida	3.69E-05	2.18E-06	1.47E-06	4.61E-05	9.17E-06	9.42E-05	8.16E-06
K01190	beta-gala	Other oligosaccharide degra	step1_Organic_carbon_oxida	0.000577	0.000132	0.000244	0.000448	0.000292	0.000109	2.25E-05
K01195	beta-gluc	Hemicullulose debranching	step1_Organic_carbon_oxida	1.14E-05	9.05E-06	1.63E-05	0.000106	6.68E-05	0	1.42E-06
K01198	beta-xylos	Other oligosaccharide degra	step1_Organic_carbon_oxida	0.000441	0.000463	0.000455	0.000176	0.000139	3.60E-05	9.63E-06
K15229	mauB	Methyl amine -> formaldehy	step1_Organic_carbon_oxida	0	0	1.22E-06	0	0	0	0
K15228	mauA	Methyl amine -> formaldehy	step1_Organic_carbon_oxida	0	0	1.22E-06	0	0	1.12E-06	0
K00121	frmA	Formaldehyde oxidation	step1_Organic_carbon_oxida	0.000582	9.16E-05	0.000103	0.00017	8.16E-05	0.000859	0.000522
K00123	fdoG	Formate oxidation	step1_Organic_carbon_oxida	0.000291	0.001182	0.001499	0.001329	0.001219	0.001087	0.000503
K00124	fdoH	Formate oxidation	step1_Organic_carbon_oxida	0.000112	0.000465	0.000536	0.000555	0.000332	0.000423	0.000375
K00125	fdhB	Formate oxidation	step1_Organic_carbon_oxida	0	0.000188	0.00023	0.000272	0.000527	0.000226	7.27E-05
K03381	catA	Protocatechuate/Catechol de	step1_Organic_carbon_oxida	9.44E-05	9.85E-08	1.75E-06	9.79E-06	1.32E-06	7.14E-05	0.000404
K01779	cellulase	Cellulose degrading	step1_Organic_carbon_oxida	0.000259	0.00018	0.000153	0.000139	5.97E-05	0.000147	0.000165
K00148	fdhA	Formaldehyde oxidation	step1_Organic_carbon_oxida	0.000128	4.56E-06	5.62E-06	3.86E-05	1.99E-05	0.000206	1.19E-05
K00625	pta	Acetogenesis	step6_Fermentation	0.000478	0.000362	0.000434	0.000309	0.00014	4.06E-05	8.54E-05
K00169	porA	Pyruvate oxidation	step6_Fermentation	1.66E-06	0.000348	0.000352	0.00059	0.000802	0.000295	7.17E-05
K03518	coxS	Aerobic CO oxidation	step1_Organic_carbon_oxida	0.000142	0.000317	0.000625	0.000207	5.01E-05	0.000291	0.001017
K03519	coxM	Aerobic CO oxidation	step1_Organic_carbon_oxida	1.30E-05	0.000143	0.000292	0.000118	3.07E-05	9.42E-05	0.000643
K14468	mcr	3 Hydroxypropionate cycle	step2_Carbon_fixation	7.82E-06	0	1.64E-06	0	0	0	0
K14469	K14469	3 Hydroxypropionate cycle	step2_Carbon_fixation	0	0	3.04E-07	0	0	0	0
K14466	K14466	3HP/4HB	step2_Carbon_fixation	0	6.89E-06	2.24E-05	3.76E-05	0.000173	2.70E-05	2.24E-05
K00249	acyl-CoA c	Fatty acid degradation	step1_Organic_carbon_oxida	0.000345	0.000184	0.000213	0.000277	0.000214	0.00288	0.001294
K00001	adh	Alcohol utilization	step6_Fermentation	0.000725	0.000295	0.000347	0.000316	0.00015	0.000272	0.000793
K01200	pullulanase	Amylolytic enzymes	step1_Organic_carbon_oxida	0.000142	9.79E-05	0.000151	0.000132	4.19E-05	7.63E-06	6.53E-06
K01207	hexosamin	Chitin degrading	step1_Organic_carbon_oxida	0.00024	0.000155	0.000276	0.000263	0.000104	0.000193	0.000155
K01209	arabinosid	Hemicullulose debranching	step1_Organic_carbon_oxida	0.0003	0.000405	0.000335	0.000353	0.000182	0.000108	2.55E-05
K10713	fae	Formaldehyde oxidation	step1_Organic_carbon_oxida	0	0.00039	0.000399	0.000379	0.000495	0.000307	6.81E-05
K01601	Form II	CBB cycle - Rubisco	step2_Carbon_fixation	0	0.000384	0.000337	0.000397	0.000494	0.000149	0.000142

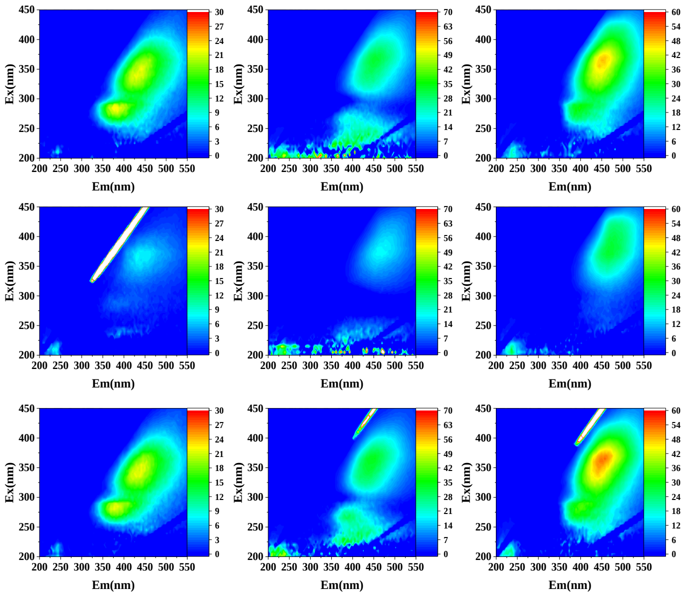

35-1\35-2\35-3 DOM

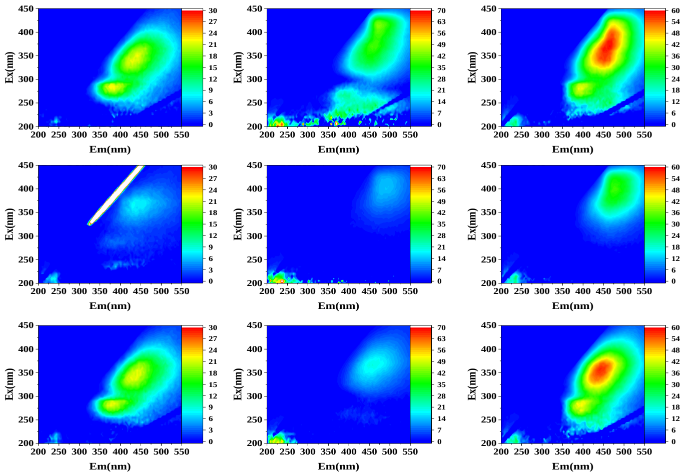

55-1\55-2\55-3DOM

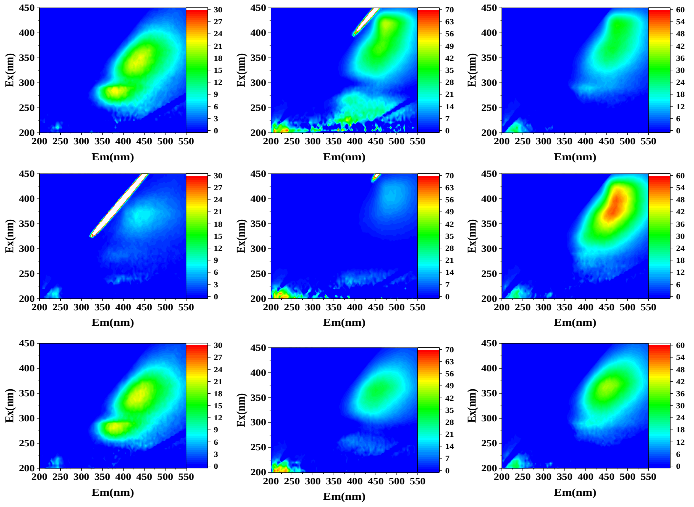

75-1\75-2\75-3DOM

Figure S1 3D-EEM of DOM variation during operational process.

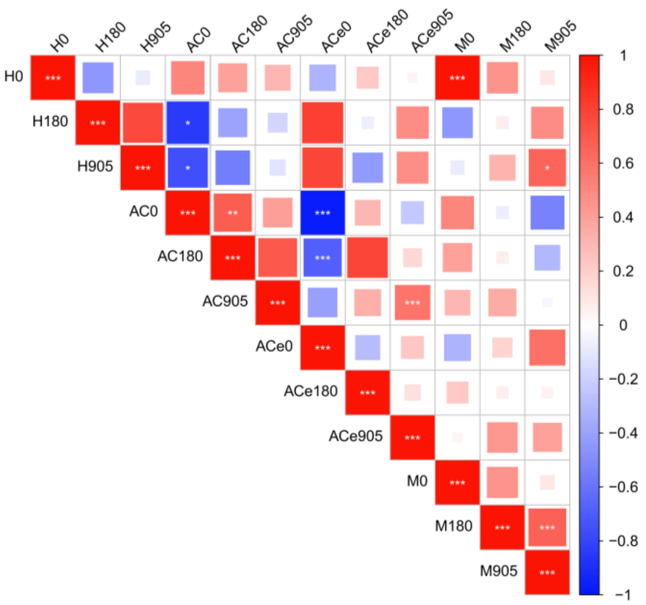

Figure S2 Correlation of the four phases of each test over time. * represents $p < 0.05$,** represents $p < 0.01$, *** represents $p < 0.001$.

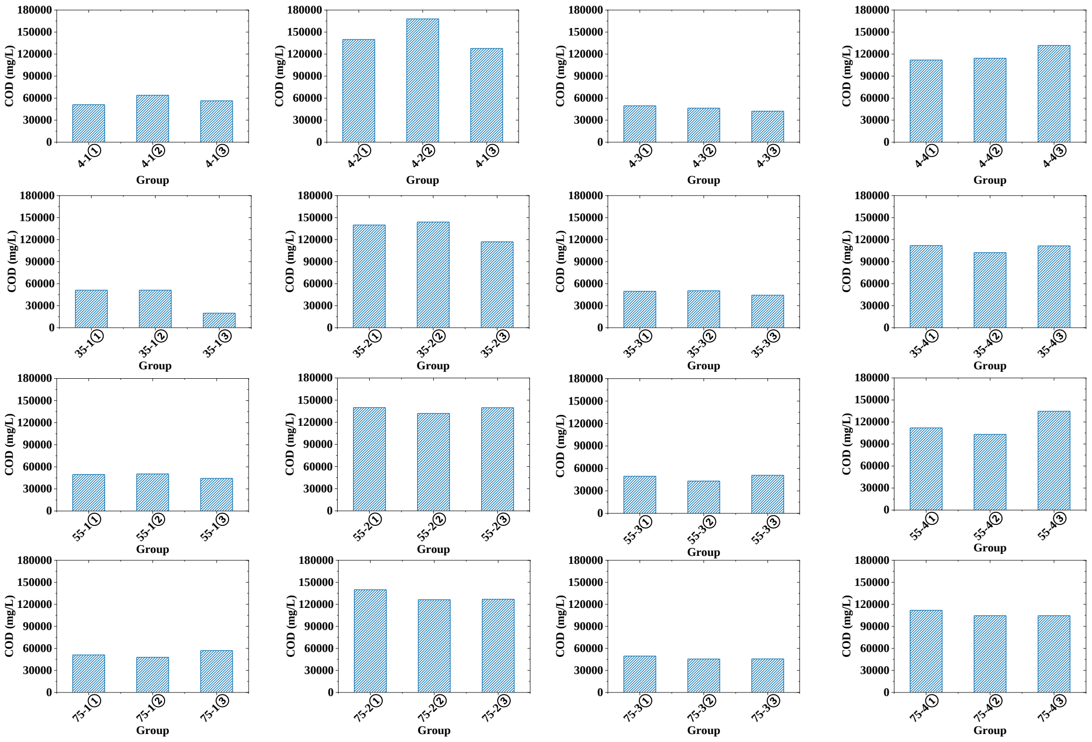

Figure S3 The COD distribution of the tests.

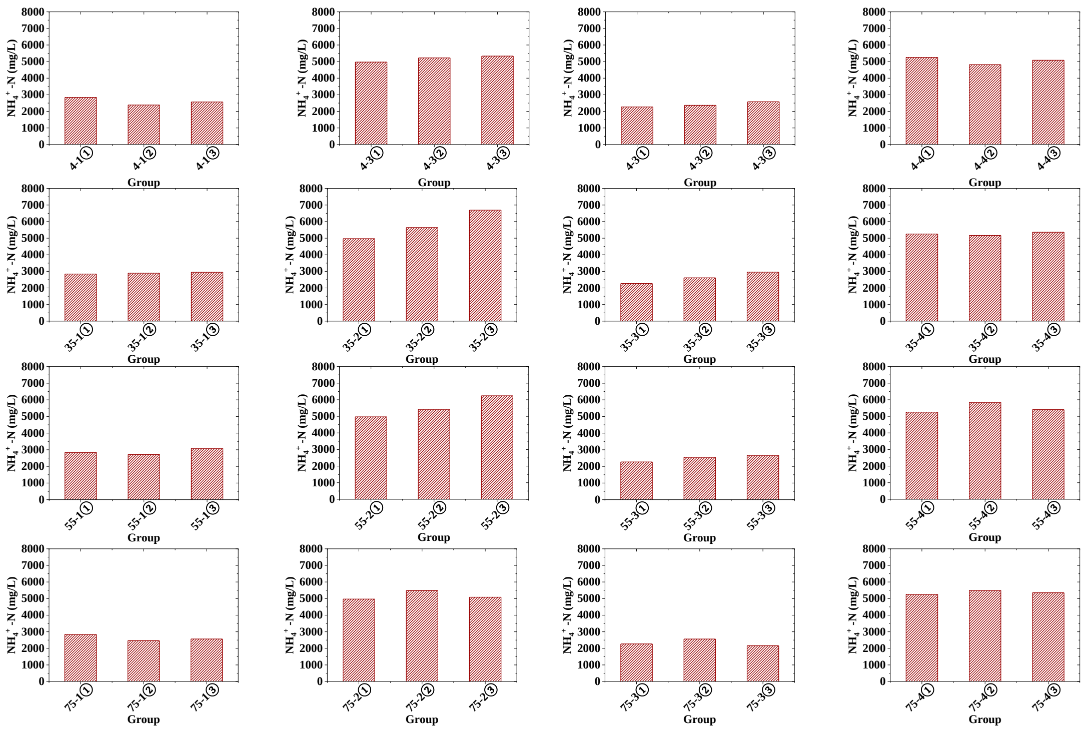

Figure S4 TAN concentration of each testes

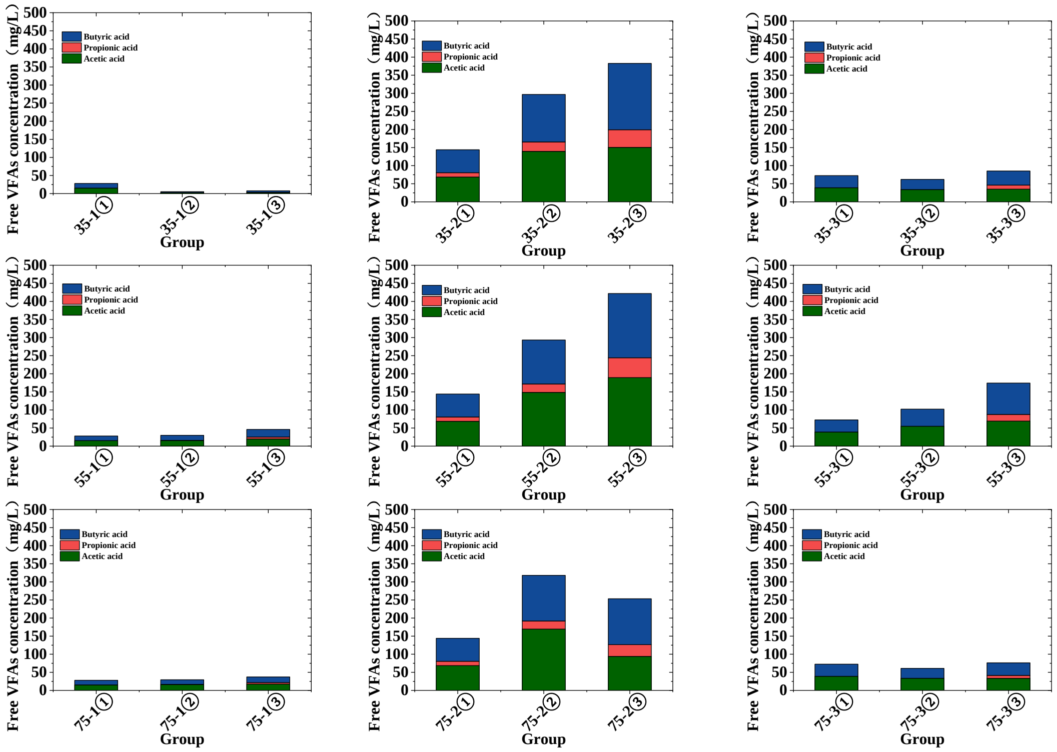

Figure S5 FVFA calculation of different temperature tests.

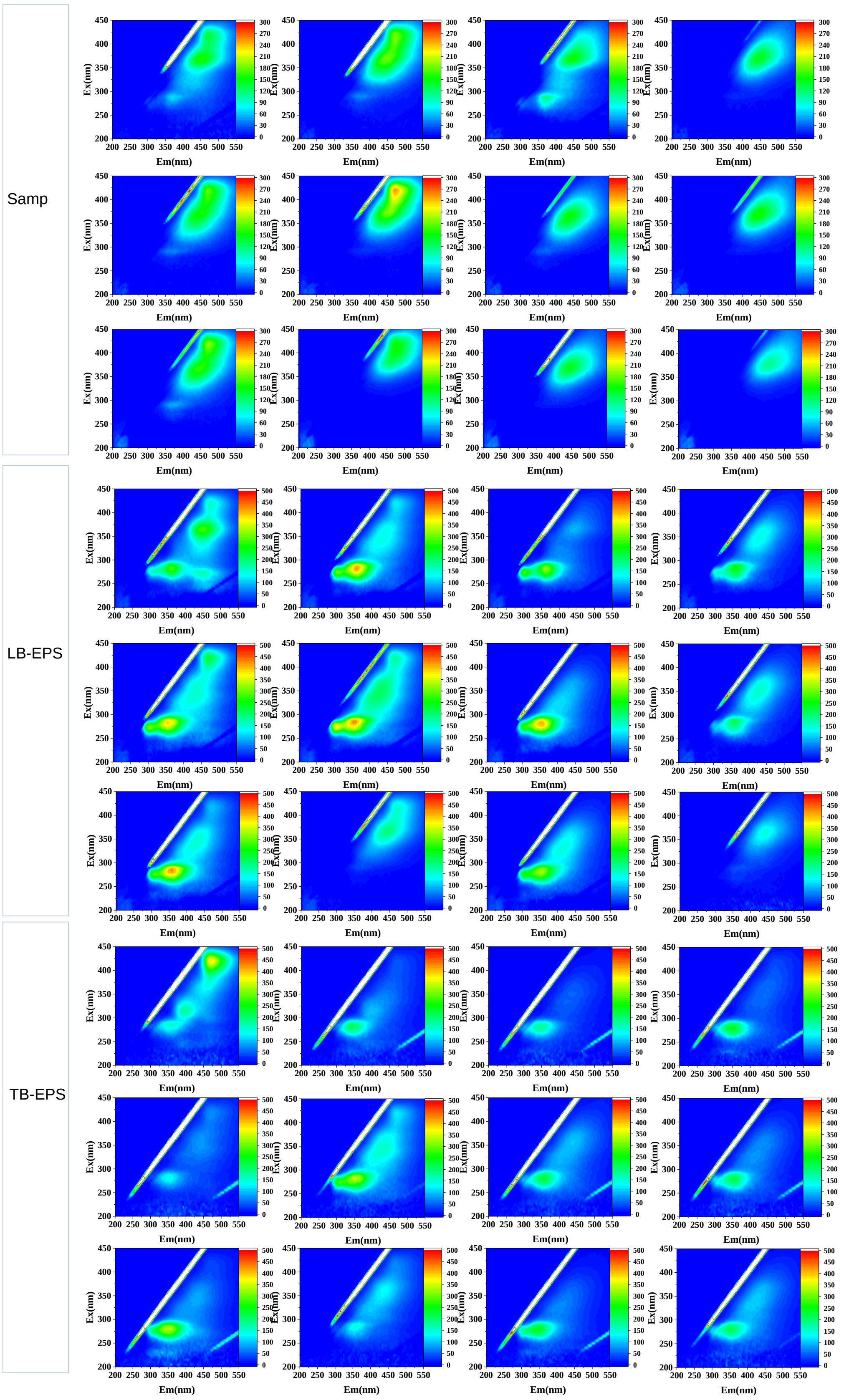

Figure S6 3D-EEM analysis of each kinds of EPS.

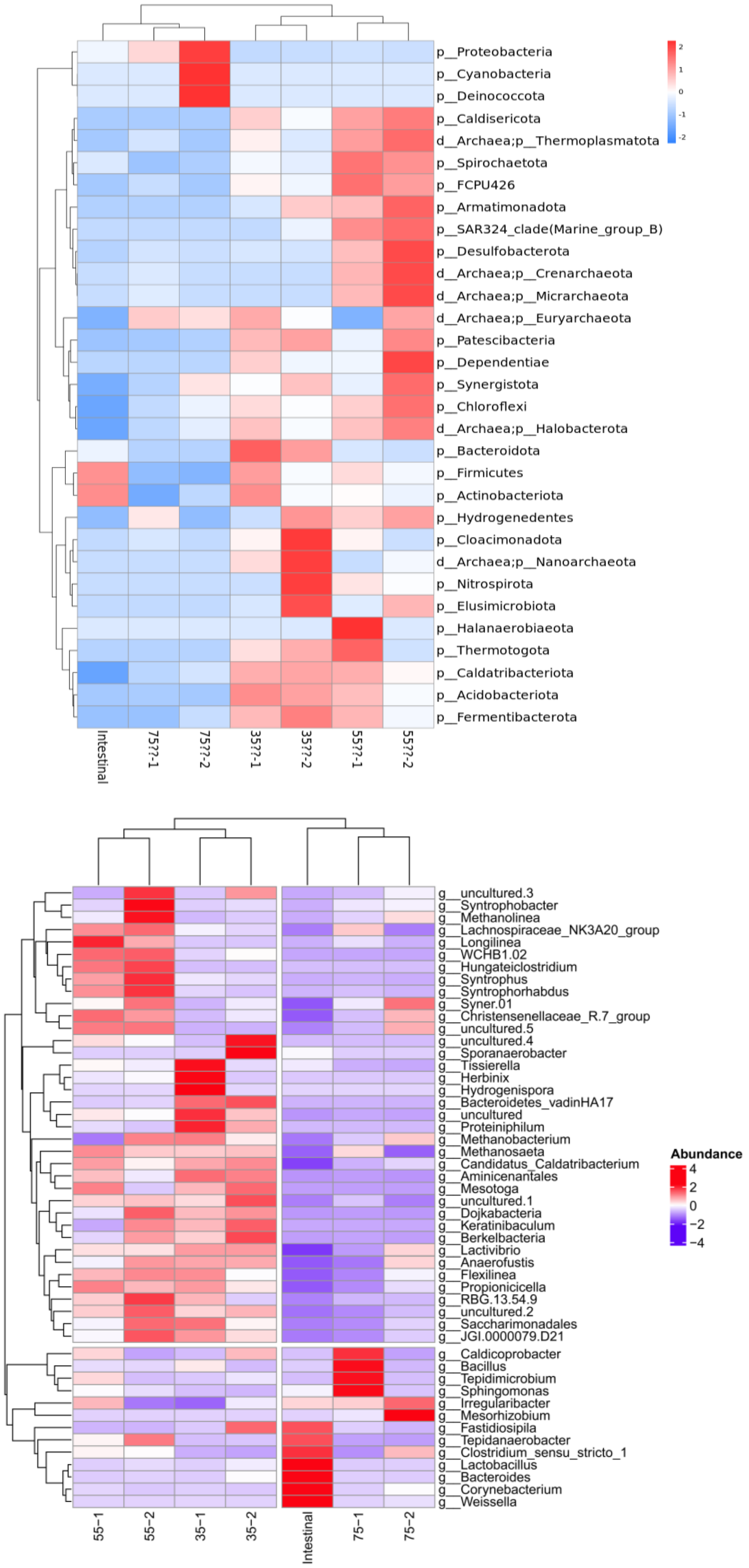

Figure S7 Heatmap of all phyla (a) and the top 50 genera of the microbial community

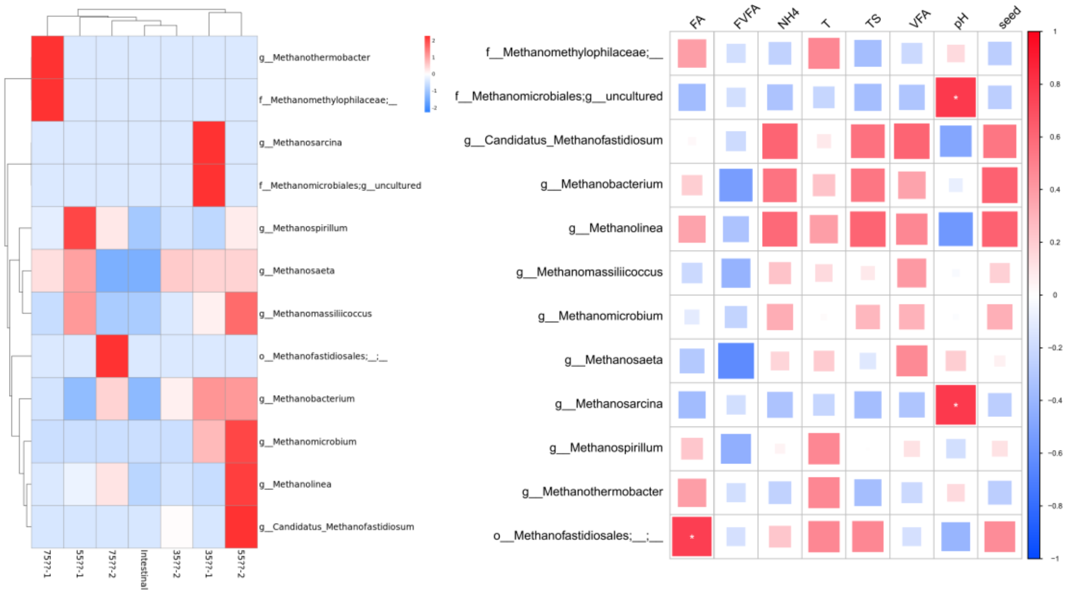

Figure S8 Heatmap of Methanogens in genus level and the correlation.

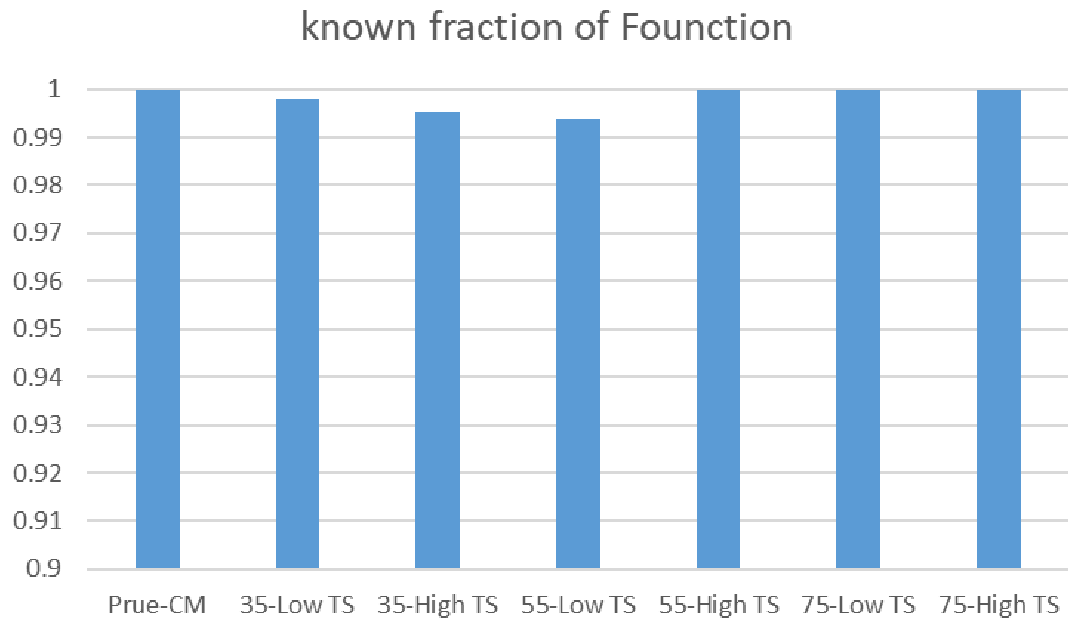

Figure S9 The accuracy of functional prediction by Tax4Fun for each sample.

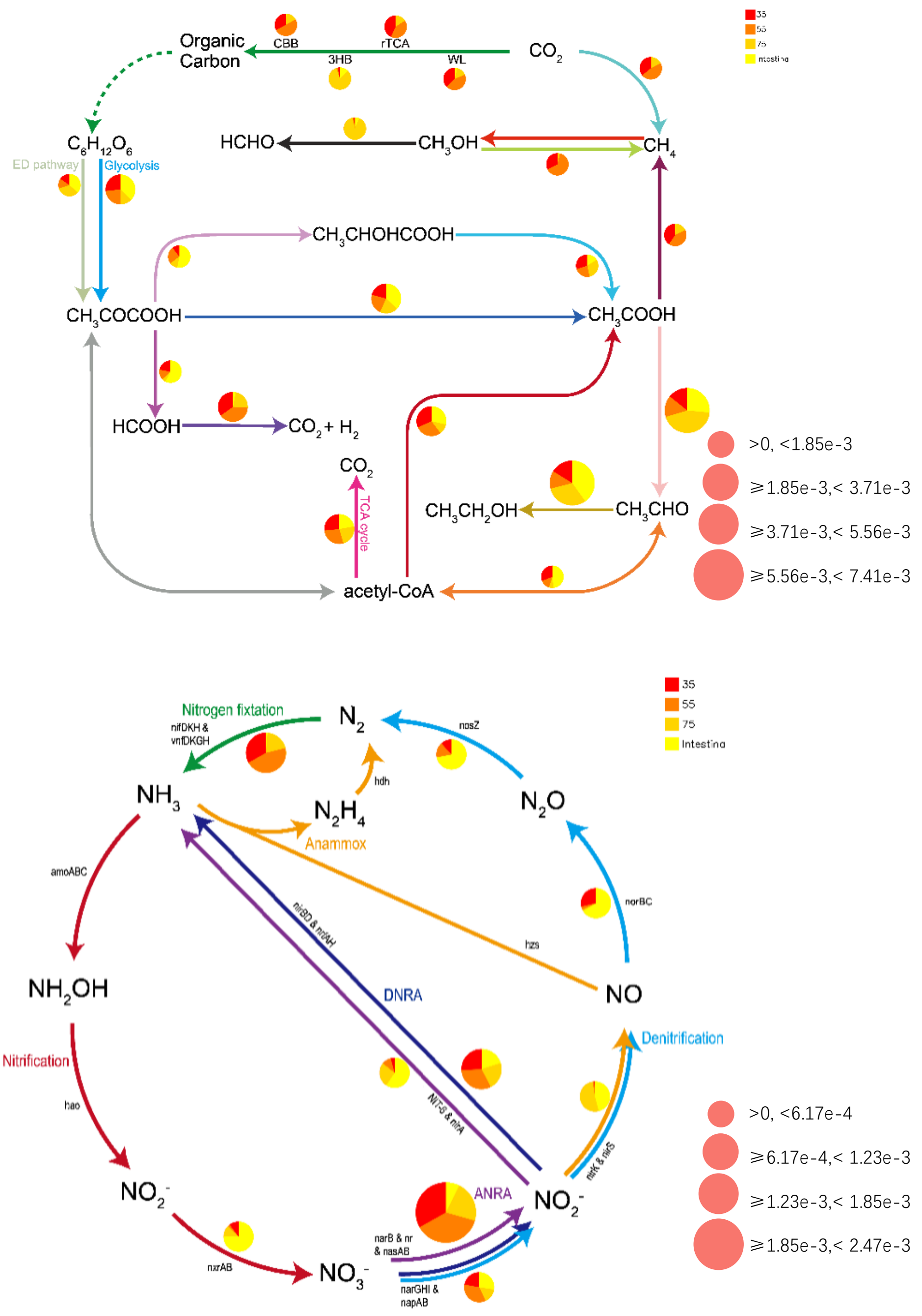

Figure S10 Abundance of sulfur cycle-related genes for community function prediction.

This figure was drawn using an online website. Our gratitude goes to this website：https://www.cloudtutu.com.
